# Effect of extreme temperature changes on phenolic, flavonoid contents and antioxidant activity of tomato seedlings (*Solanum lycopersicum* L.)

**DOI:** 10.7717/peerj.11193

**Published:** 2021-05-12

**Authors:** Haifa A.S. Alhaithloul, Fatma H. Galal, AlaaEddeen M. Seufi

**Affiliations:** 1 Biology Department, College of Science, Jouf University, Sakaka, Aljouf, Saudi Arabia; 2 Department of Entomology, Faculty of Science, Cairo University, Giza, Greater Cairo, Egypt; 3 Department of Basic Sciences, Deanship of Common First Year, Jouf University, Sakaka, Aljouf, Saudi Arabia

**Keywords:** *Solanum lycopersicum*, Tomatoes, Climatic changes, Phenolic content, Flavonoid content, Thermal sterss, Antioxidant activity

## Abstract

**Background:**

Climatic changes are the most important abiotic factor affecting plant growth, crop quality and nutritional value. Plants exposed to thermal stress respond by accumulation of secondary metabolites/molecules (SMs). Tomato (*Solanum lycopersicum*) is a cosmopolitan crop, eaten by most of the world’s people because it is highly nutritious plant. It is cultivated in more than 16 thousand hectares in Saudi Arabia and thus is influenced by extreme climatic changes.

**Objective:**

In the current study, the phytochemical effect of thermal stress was investigated in seedlings of *S. lycopersicum*. Such information will be very helpful in developing more tolerant tomato cultivars in a climate change scenario.

**Methods:**

Seedlings of *S. lycopersicum* were subjected to heat shock; HS1 and HS2 (45 and 50 °C) and cold shock; CS (4 °C) in comparison to control; Con (25 °C). Phenolic compounds, flavonoids, total phenolic content (TPC), total flavonoid content (TFC) and antioxidant activity were estimated under the four temperature treatments.

**Results:**

Using 23 standards (17 phenolic and six flavonoids), HPLC resulted in the estimation of 16, 20, 15 and 18 compounds for Con, CS, HS1 and HS2, respectively. Differences in the amounts of total phenolics, and total flavonoids were strongly correlated to thermal stress. CS plants exhibited the highest number of signals and the highest absolute quantities of total phenolics, flavonoids and sum of both. The major peaks of phenolics were (Chlorogenic acid, Resvertol), (Vanillic acid, Benzoic acid, Quinol), (Vanillic acid, Benzoic acid) and (Vanillic acid, Benzoic acid) for Con, CS, HS1 and HS2, respectively. The major peaks of flavonoids were (Quercetin, Myricetin), (Quercetin, Rutin), (Quercetin, Rutin, Catechin) and (Quercetin) for Con, CS, HS1 and HS2, respectively. CS plants contain the highest amounts of Benzoic acid (8010.37 mg/kg FW) and Quercetin (2319.48 mg/kg FW). The highest TPC (131 mg GAE/100 g FW) and TFC (61 mg QE/100 g FW) were determined in the case of CS plants. In terms of IC_50_s, the CS plants showed the highest antioxidant activities (lowest values) in both of DPPH (467.73 µM TE/100 g FW) and ABTS (8.97 µM TE/100 g FW) assays.

**Conclusions:**

Our findings supported that the complexity and quantity of phenolics and flavonoids in tomato’s extract are strongly related to thermal stress. Additionally, the CS plants demonstrated more desirable phytochemical profile over the other treatments. CS plants exhibited higher number, absolute amounts of SMs, higher TPC and TFC than those of Con, HS1 and HS2 plants. Additionally, CS plants showed higher antioxidant activity than that of both HS1 and HS2 plants. Such results are very useful in justifying mechanism of tolerance in tomato plant to thermal stress in the context of climate change. Additional research has turned on to reveal molecular response of tomato to such thermal stress.

## Introduction

Because of their important bioactive compounds, several plants are seen as valuable natural resources. The multiple uses of plants as food, health promoting agents, food additives and supplements have led to increasing studies to throw light on contents of their bioactive compounds (Kroymann, 2011; Yang et al., 2018; Arnold, Kruuk & Nicotra, 2019). A lot of published research has reported that production of SMs is a defense mechanism of plants against different types of stress (Isah, 2019). In addition, temperature changes may affect plant metabolism in several ways including SMs production (Morison & Lawlor, 1999). For instance, thermal stress created oxidative stress in the somatic embryos of *Eleutherococcus senticosus* resulting in increasing SMs production (Shohael et al., 2006).

With the growing demand for food and for overwhelming the crop losses resulting from climate changes including global warming, there is an urgent need to develop strategies for enhancing crop productivity (Ainsworth & Ort, 2010). The tomato plant, *Solanum lycopersicum* L., is regarded the second most important vegetable plant worldwide, after the potato, *Solanum tuberosum* L. (FAOSTAT, 2019). It belongs to the family Solanaceae and formerly it was called *Lycopersicon esculentum* Mill. The annual yield of tomato is approximately 188 million tons increasing by an average of 2.9% per year (FAOSTAT, 2019). In order to ensure prime quality and high yield, tomato prefers dry and cold growing conditions. Although the optimum growing temperature of most tomato varieties lies between 21 and 25 °C, the plants ensure their survival within flexible range of temperature (10–38 °C). Due to its high adaptation capacity to different growing conditions, it is cultivated around the world in open fields and in greenhouses, as well (Camejo et al., 2005). In addition to its commercial and economic values, tomatoes offer such balanced and healthy diet that it is consumed in different ways and in several dishes. It is rich in vitamins, minerals, sugars, essential amino acids and fibers (Hedges & Lister, 2007). Another advantage for tomato is being considered a model plant as it possesses diverse developmental traits (Emmanuel & Levy, 2002; Sun et al., 2006; Carvalho et al., 2011).

Over to its scientific, economic and nutritional values, tomatoes like several plants have been obliged to evolve flexible mechanisms for conciliation of dramatic, seasonal climatic and environmental changes (e.g., light, temperature, humidity, etc.). Numerous plants have been reported to produce a stockpile of SMs in response to changes of micro- and macro-environmental conditions (Morison & Lawlor, 1999; Shohael et al., 2006; Kroymann, 2011; Berini et al., 2018). Biosynthesis of these molecules is considered an adaptive power of a plant to cope with stressful conditions (Edreva et al., 2008). For instance, the brown pigments resulting from condensation of proteins and chlorogenoquinone reduced stress damage in tobacco; accumulation of polyamines in water stressed tobacco and formation of phenylamides were observed in heat shocked bean (Edreva et al., 2008). Temperature and light quality impacted ginsenosides’ production in *Panax ginseng* hairy root cultures (Yu et al., 2005). Plants and their bioactive compounds are directly consumed or used to protect against many diseases (De Luca et al., 2012; Wurtzel & Kutchan, 2016). Previous studies have clarified that SMs are synthetized by plant to protect them against stressful environment, insects, pathogen and other herbivores’ attacks (Hartmann, 2004; Freeman & Beattie, 2008; Kim, Choi & Sano, 2010). Consequently, biotic and abiotic elicitors have been used to improve SMs production. Several studies have reported the role of SMs in suppressing plant oxidative stress (Sato et al., 2006; Shohael et al., 2006; Poschenrieder et al., 2008; Hänsch & Mendel, 2009; Goyal et al., 2012; Selmar & Kleinwächter, 2013). Cultivating tomatoes under moderate thermal stress resulted in significant decrease in pollen viability, the number of pollen grains released and the number of fruit set. Lower fructose and glucose contents but higher sucrose content in the androecium were also observed (Sato et al., 2006). Some researchers have investigated the effect of moderate thermal and other stresses on tomato plants. For example, drought stress resulted in higher α-tocopherol content in *S. chilense* than in *S. lycopersicum* (Loyola et al., 2012). In addition, three tomatoes cultivars (“Arvento”, “LA1994” and “LA2093”) were investigated for heat and drought stress. Significant decreases in dry weight of shoot, carotenoid, chlorophyll A and starch contents in “Arvento” under thermal stress were reported in comparison to control and other cultivars (Zhou et al., 2017). Additionally, the accumulation of reactive oxygen species (ROS) and induction of oxidative stress are reported common features in tomatoes at single drought and thermal stress (LutforRahman et al., 2000; Camejo et al., 2006; 2007). A deep understanding of tolerance of tomatoes against heat and cold shocks is thus essential for servicing and developing future crop systems. Understanding the variations of SMs and scavenging system of tomato subjected to heat and cold shocks may help in developing more desirable tolerant tomato cultivars. However, the studies concerning thermal stress in tomato plants are insufficient (*e.g.,* Rivero et al., 2001; Scarano et al., 2020).

Therefore, the main question of the research was to test the possibility that thermal stress may increase secondary metabolites which could be useful to make plants more tolerant to extreme climatic events. Tomato seedlings, *S. lycopersicum* L., were used to perform all analyses because young plants could be strongly affected by these extreme events resulting in severe consequences on the development and crop yield. Herein, thermally-stressed tomato seedling extracts were estimated for phenolic and flavonoid compounds by using HPLC technology. Additionally, the antioxidant activities were investigated by DPPH and ABTS assays.

## Methods

### Plant materials

Seeds of cultivar GS12 F1 of the tomato, *Solanum lycopersicum* L., were obtained commercially from Nur-Sultan company (AstanaAgroA, Kazakhstan). Seeds were sterilized and germinated in 7.5 cm pots with moist compost which was covered by a thin layer of vermiculite and pots were covered with a cling film until germination. After 2 weeks, once 2–3 cm tall, seedlings were transplanted into 30 cm pots containing moist multi-purpose compost. Seeds were grown for 28 days in a growing chamber under highly controlled conditions (25 ± 1 °C, 50–60% RH, 10 h Dark: 14 h Light photoperiod, with approximate light intensity of 450 μmol m^−2^s^−1^). The plants were irrigated with 25 mL of nutrient solution every day. At the day 28 post-seedling, all visually healthy, homogenous growing plants were transplanted to new vermiculite pots, individually, to encourage building up of stronger and bigger root system. These new pots were maintained in the same growing chamber for additional 7 days to allow having deeper and massive roots. Additionally, this period helped plants to acclimatize and to become more ready for experimental stress.

### Experimental design

Forty pots of approximately 35 days-old plants were divided into four groups (10 pots/each). The first group was grown at 25 °C in the growing chamber and used as control **(Con)**. The second group was exposed to cold-shock at 4 °C for 1 h/day for a period of 7 days **(CS)**. The third group was exposed to the first level of heat-shock at 45 °C for 1 h/day for a period of 7 days **(HS1)**. The fourth group was exposed to the second level of heat-shock at 50 °C for 1 h/day for a period of 7 days **(HS2)**. Heat and cold shocks were provided by keeping plants into digital incubator (Thomas Scientific Inc., USA) which was previously set up at the requested temperature and period. Before and after cold or heat exposure period, plants were grown in the controlled growing chamber. Each experimental group had three biological replicates.

### Preparation of the extract for analyses

The whole plants of the four groups were collected immediately after the end of the stress (42 days-old) and then freeze-dried at −80 °C for maintaining quality and for avoiding loss of compounds. The epigeal part (fruits were not included) of freeze-dried plants were ground into powder by using laboratory mill. About 100 g of the powder of each biological replicate of the four treatments were soaked in 200 mL of 80% ethanol with stirring at room temperature for 3 days. The mixtures were filtered, separately, through Whatman #1 filter paper and the filtrates of 12 replicates (4 experimental groups * 3 replicates) were concentrated in a speed vacuum (Modulspin 31, Biotron, Seoul, Korea). Crude ethanol extracts of the four experimental groups were stored in sealed dark vials at 4 °C until further use.

### Chemicals and kits

All chemicals, trolox (6-hydroxy-2,5,7,8-tetramethychroman-2-carboxylic acid), gallic acid, quercetin, DPPH (2,2-diphenyl-1-picrylhydrazyl), ABTS (3-ethylbenzothiazoline-6-sulfonic acid), solvents for extraction and sample preparation, 23 standards of phenolic and flavonoid compounds were purchased directly from Sigma–Aldrich (Saint Louis, MO, USA). Folin-Cioceltau’s reagent was purchased from Merck (Darmstadt, Germany).

### High performance liquid chromatography (HPLC) analysis

The ethanolic extracts of three biological replicates per experimental group and 23 standards (Pyrogallol, Quinol, Gallic acid, Catechol, p-Hydroxy benzoic acid, Chlorogenic acid, Vanillic acid, Caffeic acid, Syringic acid, p-Coumaric acid, Benzoic acid, Ferulic acid, Ellagic acid, o-Coumaric acid, Resvertol, Cinnamic acid, Rosemarinic acid, Quercitin, Catechin, Rutin, Neringein, Myricetin and Kampherol) were admitted to quantitative analyses by HPLC, separately. All standards were dissolved in 1 mL of methanol, filtered through Whatman Nylon Membrane Filter and then injected into the column. Agilent 1260 Infinity HPLC Series (Agilent Technologies, USA), connected to a quaternary rapid pump, a Zorbax Eclipse plus C18 column (100 mm × 4.6 mm i.d.) and VWD detector set at 284 nm was operated at 30 °C. A ternary linear elution gradient using (A) HPLC grade water 0.2% H_3_PO_4_ (v/v), (B) methanol and (C) acetonitrile was operated for separation. 20 μL of each sample was injected. The analyses were performed using the above mentioned standard compounds. Extracts of three biological replicates of each experimental group were analyzed by HPLC. Compounds were identified according to similarity of retention time (RT) and spectra of 23 standards. Concentrations of a compound in mg/kg were computed using the standard curve of the corresponding standard in each temperature treatment.

### Total phenolic content (TPC)

A modified Folin–Ciocalteu method (Lin & Tang, 2007) was followed to estimate the concentration of phenolic compounds in our extract samples (diluted to 1 mg/mL). One mL of Folin Ciocalteu reagent was added to 1 mL of each extract (1 mg/mL). Three min later, 1 mL of 10% Na_2_CO_3_ solution and 7 mL of distilled H_2_O were added. Mixture was vortexed for 15 s and then allowed to stand and kept for 90 min in dark for color development. Absorbance was then measured at 760 nm using a Shimadzu-UV-1201 spectrophotometer (Kyoto, Japan). Extracts of three biological replicates of each experimental group were analyzed and a standard curve was constructed using gallic acid. Concentrations of the total phenolic compounds were expressed in mg of gallic acid equivalents per 100 g fresh weight (FW) (mg GAEs/100 g FW).

### Total flavonoid content (TFC)

Zhishen, Mengcheng & Jianming (1999) method was employed to estimate the total flavonoid content in our extracts. One mL of the extract was diluted with four mL of distilled H_2_O and 0.3 mL of 5% NaNO_2_ (w/v) was added. Five min later, 0.3 mL of 10% AlCl_3_ (w/v) was added. After 6 min, 2 mL of 1 M NaOH and 2.4 mL distilled H_2_O were added. Mixtures were vortexed to ensure full mixing. Absorbance was measured at 510 nm using a Shimadzu-UV-1201 spectrophotometer (Kyoto, Japan). Extracts of three biological replicates of each experimental group were analyzed and a standard curve was plotted using quercetin. Concentrations of the total flavonoids were expressed in mg of quercetin equivalents per 100 g FW (mg QEs/100 g FW).

### Antioxidant activityABTS radical cation scavenging activity assay

Samples were tested for their scavenging activity using ABTS radical cation as described by Xiao (2015). Briefly, 7 mM of ABTS were incubated in dark with 2.45 mM of K_₂_S_₂_O_₈_ for 16 h at room temperature (to generate ABTS+). Freshly prepared ABTS+ solution was diluted with ethanol until absorbance at 734 nm reads ≈0.70. Serial dilutions of each sample were prepared. 100 µL of a sample at different dilutions were thoroughly mixed with 400 µL of the ABTS+ solution. All mixtures were incubated in dark at room temperature for 6 min and absorbance was read at 734 nm. ABTS+ scavenging activity was computed according to the equation: Inhibition %= 1 – (A_sample_/A_control_) * 100

where A_sample_ is the absorbance of a sample and A_control_ is the absorbance of blank. Percentage inhibition was plotted against concentration. Results were expressed as µM trolox equivalent per 100 g FW (µM TE/100 g FW). Extracts of three biological replicates of each experimental group were analyzed.

### Determination of DPPH radical scavenging activity

Samples were tested for their scavenging activity using DPPH radical cation as described by Kim, Guo & Packer (2002). Briefly, 0.4 mM of DPPH solution and serial dilutions of each sample were prepared. A total of 400 µL of a sample at different dilutions were thoroughly mixed with 400 µL of the DPPH solution. All mixtures were incubated in dark at room temperature for 30 min and absorbance was read at 517 nm. DPPH scavenging activity was computed according to the equation: Inhibition %= 1 – (A_sample_/A_control_) * 100

where A_sample_ is the absorbance of a sample and A_control_ is the absorbance of blank. Percentage inhibition was plotted against concentration and results were expressed as µM trolox equivalent per 100 g FW (µM TE/100 g FW). Extracts of three biological replicates of each experimental group were analyzed.

### Statistical analyses

All assays were carried out in triplicates and descriptive statistics were calculated. Test of homogeneity was done to ensure insignificant difference between replicates of the same experiment. One-way analysis of variance (ANOVA) followed by post hoc tests (*Scheffè*) were done to compare between means. Significance was calculated at *P* < 0.05. Pearson’s correlation coefficient (*r*) was calculated at significance level of *P < 0.01*. In addition, dimension reduction of the 23 standard compounds was done using principal component analysis (PCA). All statistical analyses were done by using SPSS ver. 24.0 software.

## Results

### HPLC analysis

**Figure 1:**
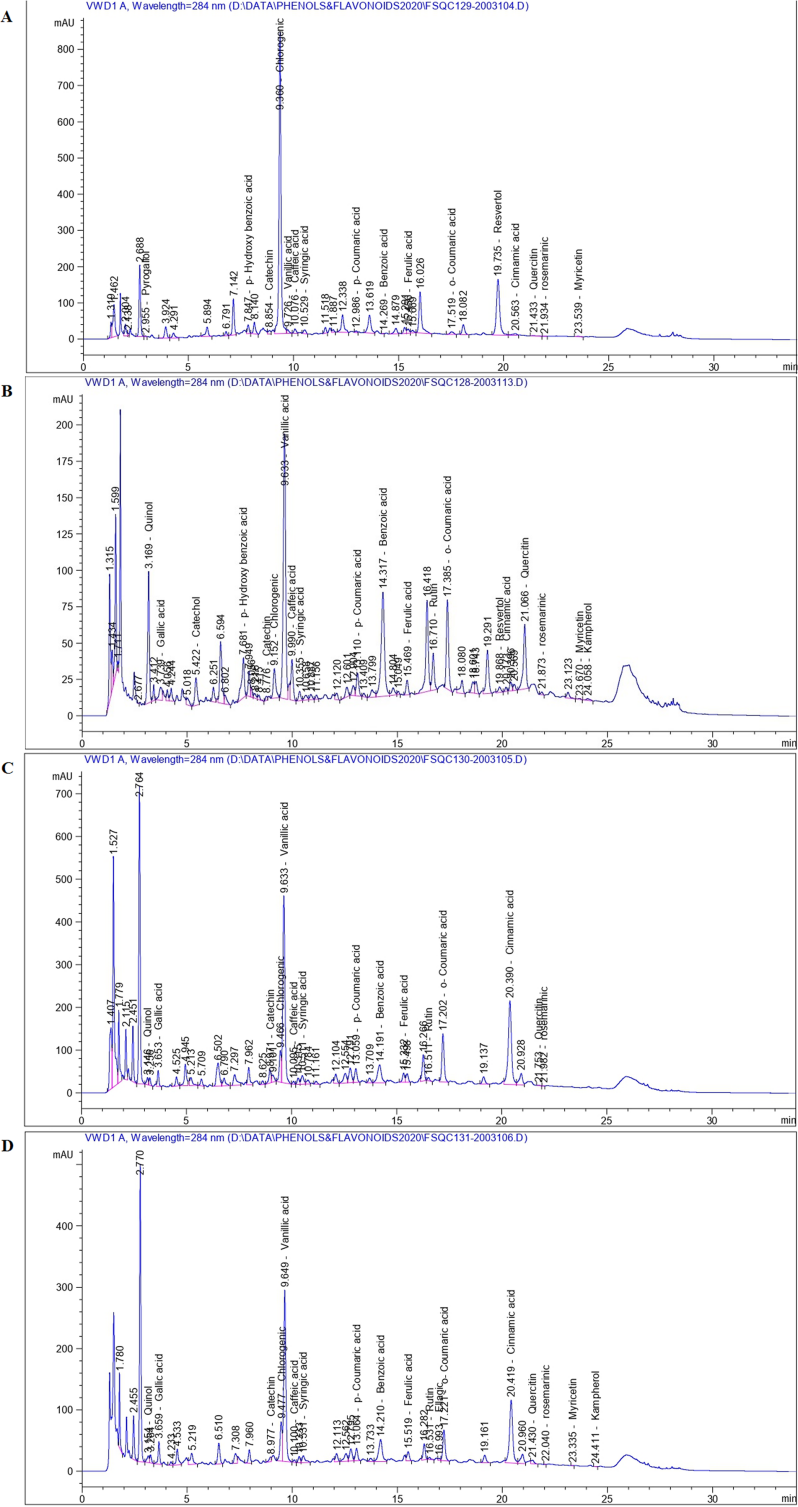
Representative HPLC chromatograms of the four ethanolic extracts of thermally stressed seedlings of *S. lycopersicum* showing all identified signals. (A) Control (Con), (B) Cold-stressed (CS), (C) First level of heat stress (HS1) and (D) Second level of heat stress (HS2).

**Figure 2:**
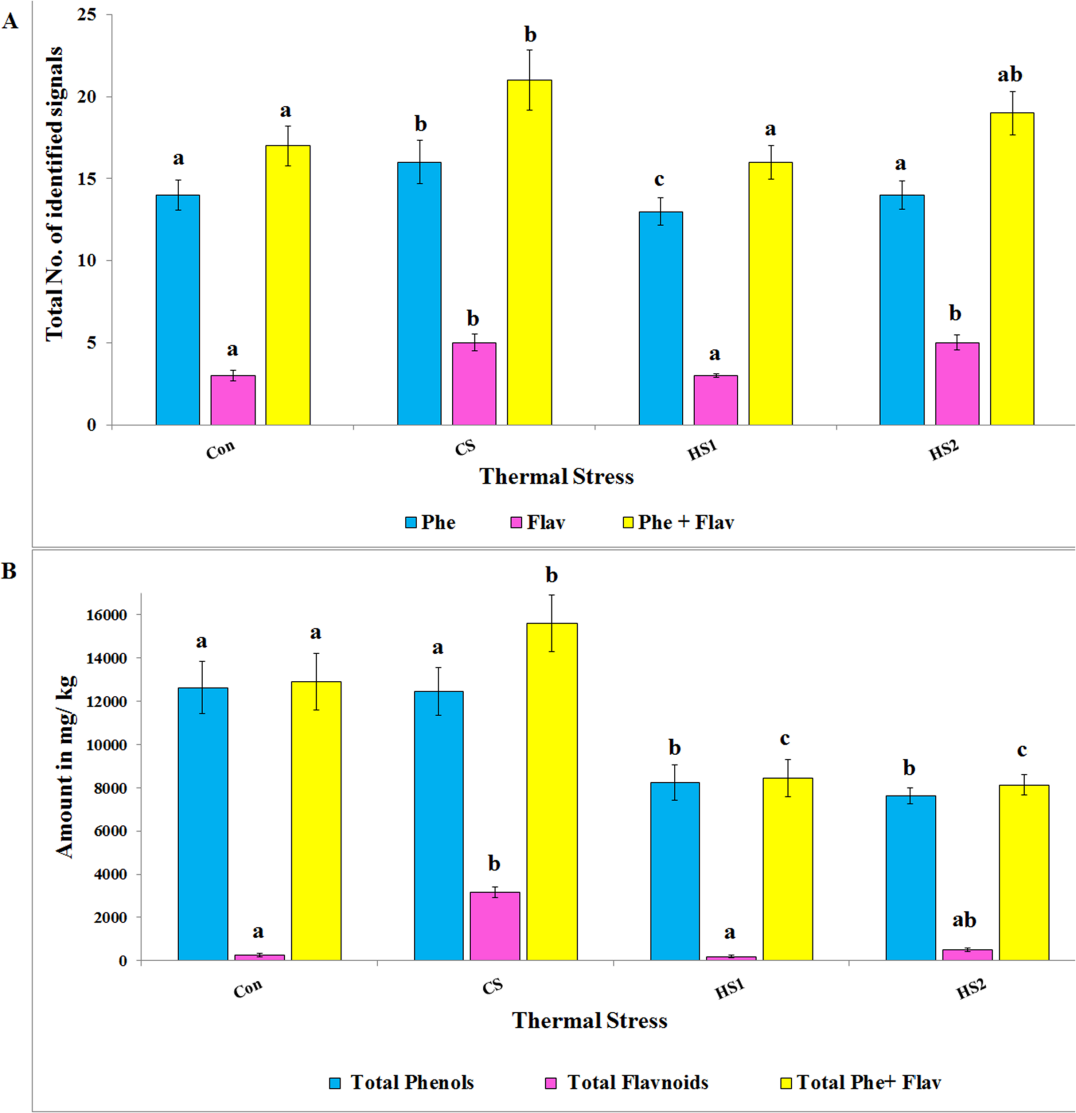
The total number of signals and amounts (mg/kg) of secondary metabolites of the 4 ethanolic extracts of thermally stressed seedlings of *S. lycopersicum*. (A) The total number of identified signals and (B) the absolute amount of phenolics, flavonoids and both. **Con, **Control; **CS,** Cold-stressed; **HS1,** First level of heat stress and **HS2,** Second level of heat stress. The lower case letters on the columns refer to significance level. Different letters refer to significant difference and the same letters refer to insignificant difference at *P < 0.05* using *Scheffè* post hoc tests.

Figure 1 displays representative HPLC chromatograms of the four ethanolic extracts of thermally stressed seedlings of *S. lycopersicum*. Based on the RT and spectra, HPLC analyses led to identification of 16, 20, 15 and 18 measurable peaks for Con, CS, HS1 and HS2 extracts, respectively. In addition, 20, 32, 26 and 21 measurable peaks failed to be identified in the cases of Con, CS, HS1 and HS2 extracts, respectively. Fig. 2A shows the total number of phenolic signals, flavonoid signals and total signals of both. One-way ANOVA clarified significant difference the number of identified phenolics (*df* = 3, *F* = 12.17, *P* = 0.002, *R*^*2*^= 0.46) with moderate positive correlation in response to thermal stress. The number of flavonoid signals (*df* = 3, *F* = 12.61, *P* = 0.002, *R*^*2*^= 0.009) and the total number of both phenolic and flavonoid signals (*df* = 3, *F* = 11.53, *P* = 0.003, *R*^*2*^= 0.16) varied significantly with weak positive correlation in regard to thermal stress. *Scheffè* post hoc tests revealed significant difference (*P < 0.001*) for all comparisons except the difference between Con and HS2 (*P > 0.05*) in the case of phenolics, between Con and HS1, CS and HS2 (*P > 0.05*) in the case of flavonoids, and between Con and HS1, Con and HS2, CS and HS2 (*P > 0.05*) in the case of total signals of both phenolics and falvonoids. Interestingly, CS plants exhibited the highest numbers of phenolics, flavonoids and total signals of both (Fig. 2A). In addition, significant difference in total absolute quantity of phenolics (*df* = 3, *F* = 34.99, *P* = 0.00, *R*^*2*^= 0.83) with strong positive correlation regarding thermal stress was observed. Significant variation was reported in total absolute quantity of flavonoids (*df* = 3, *F* = 228.31, *P* = 0.00, *R*^*2*^= 0.53) with moderate positive correlation in response to thermal stress. Meanwhile, total quantities of both phenolics and flavonoids, significantly, differ (*df* = 3, *F* = 55.04, *P* = 0.00, *R*^*2*^ = 0.91) with very strong positive correlation in regard to thermal stress (Fig. 2B). Post hoc tests revealed significant difference for all comparisons (*P < 0.001*) except the differences between Con and CS, HS1 and HS2 in the case of phenolics, between Con and HS1, Con and HS2 in the case of flavonoids, and between HS1 and HS2 in the case of both (*P > 0.05*). Again, the CS plants still displaying the highest amounts of phenolics, flavonoids and total amount of both (Fig. 2B).

Tables 1 and 2 show the total phenolic and flavonoid compounds identified by HPLC analyses of the 4 ethanolic extracts of thermally stressed seedlings of *S. lycopersicum*. Retention time of separated signals extends from 1.32 to 23.54, 1.32 to 24.0, 1.41 to 21.98 and 1.78 to 24.41 min for Con, CS, HS1 and HS2 extracts, respectively.

**Table 1:** Total phenolic compounds identified by HPLC analyses of the 4 ethanolic extracts of thermally stressed tomato seedlings, *S. lycopersicum*.

No.	Compound	RT in min				Area [mAU*s]				Area (%)				Amount (%)			
		Con	CS	HS1	HS2	Con	CS	HS1	HS2	Con	CS	HS1	HS2	Con	CS	HS1	HS2
**1**	Pyrogallol	2.96	2.9	2.9	2.9	11.789	ND	ND	ND	0.148	ND	ND	ND	0.097	ND	ND	ND
**2**	Quinol	3.17	3.17	3.15	3.15	ND*	519.664	93.633	77.134	ND	11.796	1.148	1.502	ND	7.971	1.575	1.817
**3**	Gallic acid	3.65	3.74	3.65	3.66	ND	91.146	188.64	204.735	ND	2.069	2.312	3.988	ND	0.434	0.966	1.466
**4**	Catechol	5.4	5.42	5.4	5.4	ND	149.648	ND	ND	ND	3.397	ND	ND	ND	2.53	ND	ND
**5**	p-Hydroxy benzoic acid	7.85	7.68	7.7	7.7	138.412	251.306	ND	ND	1.7382	5.704	ND	ND	2.087	4.095	ND	ND
**6**	Chlorogenic	9.36	9.15	9.47	9.48	5,567.345	169.148	477.564	443.50	69.914	3.839	5.853	8.638	29.126	0.952	2.951	3.836
**7**	Vanillic acid	9.73	9.63	9.63	9.65	79.33	1,445.71	3,192.21	2,031.868	0.996	32.85	39.12	39.576	0.673	14.658	35.621	31.692
**8**	Caffeic acid	10.08	9.99	10.1	10.1	91.67	200.685	40.669	25.315	1.151	4.555	0.498	0.493	0.263	0.613	0.141	0.126
**9**	Syringic acid	10.53	10.36	10.51	10.53	86.423	46.697	185.65	119.32	1.085	1.06	2.275	2.324	0.359	0.211	0.905	0.817
**10**	p-Coumaric acid	12.99	13.11	13.06	13.1	32.384	123.084	256.193	155.914	0.407	2.794	3.1396	3.037	0.054	0.346	0.843	0.691
**11**	Benzoic acid	14.27	14.32	14.19	14.21	3.548	813.741	549.463	431.243	0.045	18.471	6.734	8.399	0.179	64.362	47.65	52.323
**12**	Ferulic acid	15.46	15.47	15.33	15.52	96.773	75.768	153.086	118.24	1.215	1.72	1.876	2.303	0.367	0.309	0.685	0.741
**13**	Ellagic	16.9	16.9	16.90	16.99	ND	ND	ND	21.548	ND	ND	ND	0.42	ND	ND	ND	0.189
**14**	o-Coumaric acid	17.52	17.39	17.20	17.22	84.473	455.179	863.754	386.227	1.061	10.332	10.585	7.523	0.206	1.016	2.075	1.334
**15**	Resvertol	19.74	19.87	19.80	19.8	1,692.71	33.148	ND	ND	21.257	0.752	ND	ND	65.998	1.272	ND	ND
**16**	Cinnamic acid	20.56	20.17	20.39	20.42	75.056	17.605	2,158.405	1,118.244	0.943	0.3996	26.451	21.781	1.43E−01	3.34E−04	6.27E+00	4.51E+00
**17**	Rosemarinic acid	21.93	21.87	21.99	22.04	3.268	13.029	8.11954e−1	8.59627e−1	0.041	0.296	0.01	0.017	0.45	1.231	0.325	0.460

**Notes:**

* ND: Not detected.

Compounds were identified according to RT of the separated signals. Con, ****Control; CS, Cold-stressed; HS1, First level of heat stress and HS2, Second level of heat stress.

**Table 2:** Total flavonoid compounds identified by HPLC analyses of the 4 ethanolic extracts of thermally stressed tomato seedlings, *S. lycopersicum*.

No.	Compound	RT in min				Area [mAU*s]				Area (%)				Amount (%)			
		Con	CS	HS1	HS2	Con	CS	HS1	HS2	Con	CS	HS1	HS2	Con	CS	HS1	HS2
**1**	Catechin	8.85	8.78	8.97	8.98	79.97	17.900	219.50	54.59	70.70	2.87	79.00	37.05	8.39	0.14	25.36	3.08
**2**	Rutin	16.70	16.71	16.51	16.53	ND*	188.450	52.25	27.53	ND	30.23	18.81	18.69	ND	20.81	54.90	10.65
**3**	Quercetin	21.43	21.07	21.75	21.43	19.34	392.302	6.09	59.26	17.10	62.94	2.19	40.22	45.31	73.25	19.74	69.44
**4**	Neringein	22.20	22.20	22.20	22.20	ND	ND	ND	ND	ND	ND	ND	ND	ND	ND	ND	ND
**5**	Myricetin	23.54	23.67	23.48	23.34	13.81	6.79	ND	1.84	12.21	1.09	ND	1.25	46.30	2.90	ND	11.77
**6**	Kampherol	24.45	24.06	24.45	24.41	ND	17.87	ND	4.11	ND	2.87	ND	2.79	ND	2.90	ND	5.06

**Notes:**

* ND: Not detected.

Compounds were identified according to RT of the separated signals. Con, ****Control; CS, Cold-stressed; HS1, First level of heat stress and HS2, Second level of heat stress.

Two major peaks (Chlorogenic acid at RT 9.36 and Resvertol at RT 19.74) constituted more than 95% of the total amount of the tested phenolics of Con plants. However, the smallest peak of p-Coumaric acid at RT 12.9 min occupied 0.05% of the total amount of phenolics in Con plants. Very small peak for pyrogallol has been detected at RT 2.96 min. Four phenolic compounds (Quinol, Gallic acid, Catechol and Ellagic acid) were not detected in Con plants (Fig. 1 and Table 1). In parallel, two major peaks (Quercetin at RT 21.43 and Myricetin at RT 23.54) shape more than 91% of the total amount of flavonoids in Con tomatoes. Rutin, Neringein and Kampherol were not detected in Con plants (Fig. 1 and Table 2).

In the case of CS plants, three major phenolic compounds (Quinol at RT 3.2, Vanillic acid at RT 9.6 and Benzoic acid at RT 14.32 min) constituted more than 87% of the total phenolics in CS plants. Two phenolic compounds (Pyrogallol and Ellagic acid) were not detected in CS plants (Fig. 1 and Table 1). Simultaneously, two peaks (Rutin at RT 16.7 and Quercitin at RT 21.1 min) shaped more than 94% of the total flavonoids in CS plants. The flavonoid Neringein has not detected in CS plants (Fig. 1 and Table 2).

At the first level of heat shock (HS1), two large peaks were detected (Vanillic acid at RT 9.6 and Benzoic acid at RT 14.2 min). The two peaks computed about 83% of the total identified phenolics. Five phenolic compounds (Pyrogallol, Catechol, p-Hydroxy benzoic acid, Ellagic acid and Resvertol) were not detected in HS1 plants (Fig. 1 and Table 1). In the same time, Catechin at RT 8.97, Rutin at RT 16.5 and Quercetin at RT 21.75 min shaped the whole amount of flavonoids in HS1 plants. Peaks of Neringein, Myricetin and Kampherol were not detected in HS1 plants (Fig. 1 and Table 2).

At the highest tolerable heat shock in this study (HS2), two major peaks of phenolic compounds (Vanillic acid at RT 9.65 and Benzoic acidat RT 14.2 min) were detected. These peaks represented more than 83% of the total phenolic compounds in HS2 plants. Four phenolic compounds (Pyrogallol, Catechol, p-Hydroxybenzoic acid and Resvertol) were not detected in HS2 plants (Fig. 1 and Table 1). Meanwhile, the Quercetin peak at RT 21.43 min shaped more than 69% of the total identified flavonoids in the case of HS2 plants. Peak of the flavonoid Neringein has not been detected. Catechin, Rutin, Myricetin and Kampherol were detectable in moderate and small quantities in the case of HS2 plants (Fig. 1 and Table 2).

**Figure 3:**
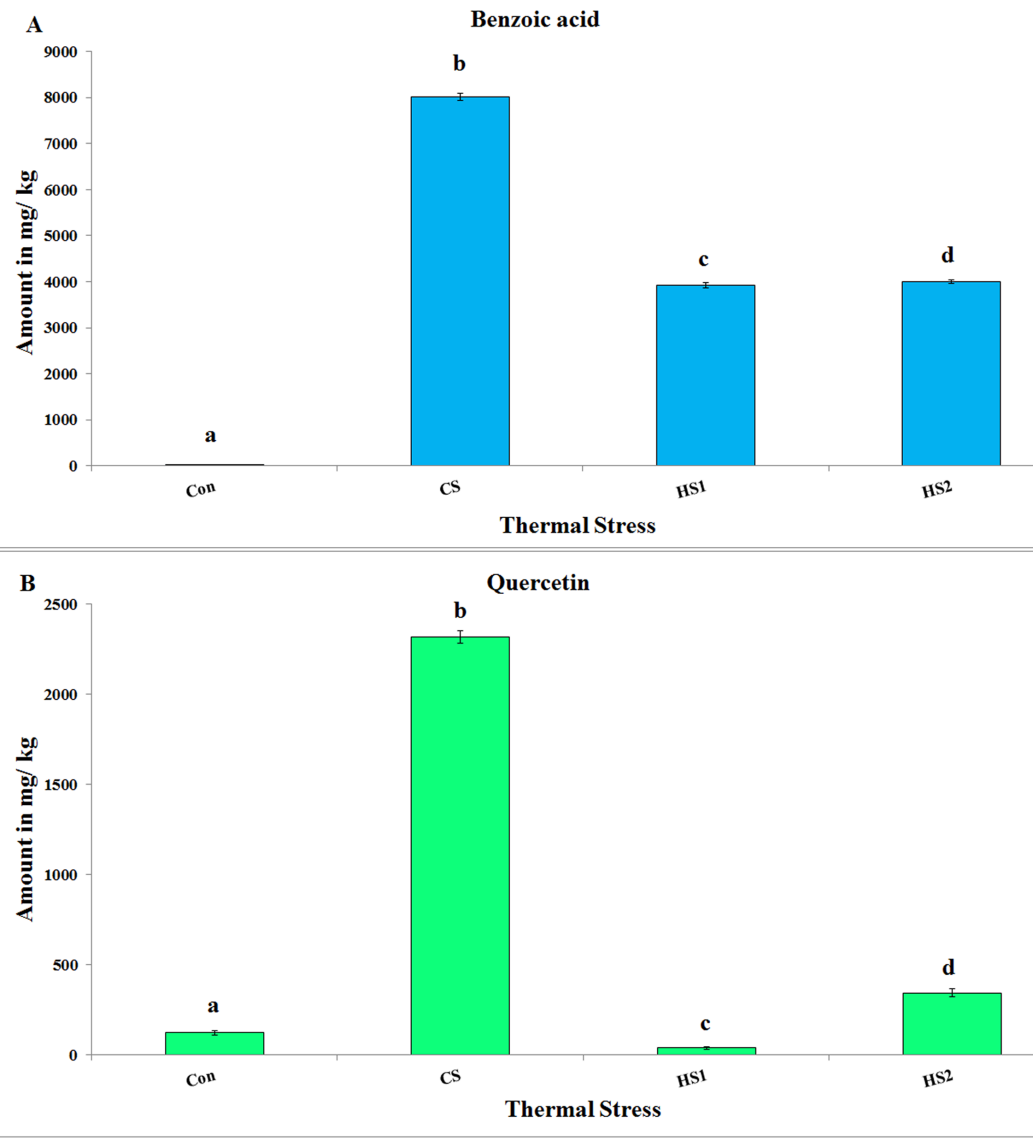
The absolute amounts (mg/ kg) of Benzoic acid and Quercetin in the 4 ethanolic extracts of thermally stressed seedlings of *S. lycopersicum*. (A) Benzoic acid and (B) Quercetin. **Con, **Control; **CS,** Cold-stressed; **HS1,** First level of heat stress and **HS2,** Second level of heat stress. The lower case letters on the columns refer to significance level. Different letters refer to significant difference and the same letters refer to insignificant difference at *P < 0.05* using *Scheffè* post hoc tests.

Remarkably, Benzoic acid (phenolic compound) and Quercetin (flavonoid compound) were commonly identified in considerable relative amounts in all treatments (Fig. 3). In addition, Benzoic acid revealed significant variance with a weak positive correlation regarding thermal stress (*df* = 3, *F* = 1261.37, *P* = 0.00, *R*^*2*^ = 0.002). Post hoc tests revealed significant difference (*P < 0.001*) for all comparisons (Fig. 3). In parallel, the amounts of Quercetin revealed significant variance with a weak positive correlation in regarding to thermal stress (*df* = 3, *F* = 375.49, *P* = 0.00, *R*^*2*^ = 0.038). Post hoc tests revealed significant difference (*P < 0.001*) for all comparisons. Excitingly, the highest amounts of both Benzoic acid (8010.374 ± 79.340) and Quercetin (2319.475 ± 34.350) were demonstrated by CS plants (Fig. 3).

One-way ANOVA of the absolute amounts of all tested compounds are summarized in Table 3. It is clear that quantities of all compounds differ, significantly, in response to thermal stress (*P* = 0.00) except for the flavonoid Neringein (*P* = 1.00). Post hoc *Scheffè* tests revealed that the quantities of all compounds differ, significantly, with thermal stress when compared to Con plants (*P < 0.001*) except for Ferulic acid of CS plants (*P > 0.05*) when compared to Con plants. All post hoc analyses are summarized in Table 4.

**Table 3:** One way ANOVA tests showing the overall significance between the amounts of phenolic and flavonoid compounds in the four ethanolic extracts of thermally stressed tomato seedlings, *S. lycopersicum*.

Compound	Sum of Squares	*df*	Mean Square	*F*	Sig.
**Pyrogallol**	341.926	3	113.975	1384.983	0.000
**Quinol**	1,869,557.932	3	623,185.977	32,653.582	0.000
**Gallic acid**	20,078.775	3	6,692.925	794.732	0.000
**Catechol**	222,672.433	3	74,224.144	9,620.546	0.000
**pHydroxy benzoic acid**	537,282.081	3	179,094.027	12,059.769	0.000
**Chlorogenic acid**	2.694E7	3	8,979,516.979	105,634.036	0.000
**Vanillic acid**	1.392E7	3	4,641,245.977	8,915.472	0.000
**Caffeic acid**	8,765.790	3	2,921.930	248.675	0.000
**Syringic acid**	3,968.975	3	1,322.992	58.800	0.000
**pCoumaric acid**	6,312.201	3	2,104.067	271.493	0.000
**Benzoic acid**	9.572E7	3	3.191E7	413,046.475	0.000
**Ferulic acid**	687.927	3	229.309	18.345	0.001
**Ellagic acid**	466.134	3	155.378	621.509	0.000
**oCoumaric Acid**	33,288.958	3	11,096.319	8,322.240	0.000
**Resvertol**	1.544E8	3	5.148E7	503,471.581	0.000
**Cinnamic acid**	577,770.404	3	192,590.135	23,344.259	0.000
**Rosemarinic acid**	30,524.399	3	10,174.800	549.989	0.000
**Catechin**	3,390.875	3	1,130.292	192.278	0.000
**Rutin**	841,292.709	3	280,430.903	6,585.462	0.000
**Quercetin**	1.056E7	3	3,518,389.203	105,026.543	0.000
**Neringein**	0.000	3	0.000	0.001	1.000
**Myricetin**	26,014.809	3	8,671.603	1,334.093	0.000
**Kampherol**	16,974.337	3	5,658.112	4,526.483	0.000

**Table 4:** Post Hoc tests (*Scheffè*) showing significant differences between the amounts of phenolic and flavonoid compounds in the 4 ethanolic extracts of thermally stressed tomato seedlings, *S. lycopersicum*.

Compound	Amount in mg/ Kg (Mean ± S.E.)			
	Con	CS	HS1	HS2
**Pyrogallol**	12.402 ± 0.320	ND	ND	ND
**Quinol**	ND*	992.032 ± 3.273^a^**	129.861 ± 2.351^b^	138.737 ± 3.034^bc^
**Gallic acid**	ND	53.982 ± 2.240^a^	79.628 ± 2.470^b^	111.926 ± 0.328^c^
**Catechol**	ND	314.878 ± 3.207	ND	ND
**p-Hydroxybenzoic acid**	263.645 ± 1.764^a^	509.701 ± 4.085^b^	ND	ND
**Chlorogenic acid**	3,678.879 ± 8.819^a^	118.470 ± 2.057^b^	243.249 ± 2.646^c^	292.972 ± 4.933^d^
**Vanillic acid**	85.021 ± 2.887^a^	1,824.286 ± 10.975^b^	2,936.314 ± 20.785^c^	2,420.427 ± 11.547^d^
**Caffeic acid**	33.206 ± 1.732^a^	76.327 ± 3.464^b^	11.637 ± 0.577^c^	9.638 ± 0.576^cd^
**Syringic acid**	45.332 ± 2.887^a^	26.279 ± 3.464^b^	74.572 ± 2.887^c^	62.369 ± 1.155^cd^
**p-Coumaric acid**	6.823 ± 0.577^a^	43.039 ± 2.887^b^	69.452 ± 0.577^c^	52.775 ± 1.155^d^
**Benzoic acid**	22.565 ± 0.560^a^	8,010.374 ± 79.340^b^	3,927.869 ± 58.210^c^	3,995.993 ± 41.110^d^
**Ferulic acid**	46.306 ± 1.155^a^	38.496 ± 1.723^ab^	56.440 ± 3.464^c^	56.562 ± 0.567^cd^
**Ellagic acid**	ND	ND	ND	14.395 ± 0.587
**o-Coumaric acid**	26.060 ± 0.577^a^	126.454 ± 0.577^b^	171.020 ± 0.882^c^	101.859 ± 0.577^d^
**Resvertol**	8,336.336 ± 11.547^a^	158.266 ± 1.732^b^	ND	ND
**Cinnamic acid**	18.014 ± 0.055^a^	4.16E−02 ± 0.577^b^	516.523 ± 5.021^c^	344.437 ± 4.910^d^
**Rosemarinic acid**	56.771 ± 1.732^a^	153.262 ± 5.774^b^	26.771 ± 1.032^c^	35.149 ± 2.309^d^
**Catechin**	22.883 ± 0.584^a^	4.540 ± 0.587^b^	49.896 ± 2.644^c^	15.277 ± 0.405^d^
**Rutin**	ND	658.833 ± 4.619^a^	108.004 ± 5.207^b^	52.808 ± 2.887^c^
**Quercetin**	123.592 ± 12.50^a^	2,319.475 ± 34.350^b^	38.825 ± 8.110^c^	344.381 ± 13.210^d^
**Neringein**	ND	ND	ND	ND
**Myricetin**	126.293 ± 3.056^a^	91.703 ± 2.008^b^	ND	58.377 ± 1.650^c^
**Kampherol**	ND	91.939 ± 0.577^a^	ND	25.088 ± 0.056^b^

**Notes:**

* ND: Not detected. All ND items are excluded from statistical analyses.

** Different letters refer to significant difference and the same letters refer to insignificant difference at *P < 0.05* using *Scheffè* post hoc tests.

Con, ****Control; CS, Cold-stressed; ****HS1, First level of heat stress and HS2, Second level of heat stress.

Pearson’s correlation analyses between the individual phenolic and flavonoid compounds are summarized in Table 5. Generally, 111 pairs exhibited significant moderate (*r > 0.5–0.6*) to strong (*r > 0.7*) correlations. Out of 111 pairs, 53 pairs exhibited positive significant strong correlations and 43 pairs exhibited negative significant strong correlations. Meanwhile, six pairs exhibited positive significant moderate correlations and nine pairs exhibited negative significant moderate correlations. The phenolic compound “Ellagic acid” exhibited positive strong correlation with only “Gallic acid” (Table 5).

**Table 5:** Summarized table of Pearson’s correlations showing the correlated pairs of the phenolic and flavonoid compounds in the 4 ethanolic extracts of thermally stressed tomato seedlings, *S. lycopersicum*.

Compound	Correlation with	*R*	Compound	Correlation with	*R*
**Pyrogallol**	Gallic acid Chlorogenic acid Vanillic acid Benzoic acid oCoumaric acid pCoumaric acid Resvertol Kampherol	−0.867** 0.998** −0.929** −0.905** −0.808** −0.877** 0.999** 0.708**	**Catechin**	Caffeic acid Syringic acid Ferulic acid Quercetin Rosemarinic acid Neringein Kampherol Cinnamic acid	−0.666* 0.812** 0.648* 0.752** −0.705* −0.703* −0.703* −0.748**
**Quinol**	Catechol pHydroxyBenzoic acid Catechin Caffeic acid Syringic acid Benzoic acid Ferulic acid Rutin Rosemarinic acid Neringein Cinnamic acid	0.990** 0.781** −0.600* 0.875** −0.725** 0.894** −0.701* 0.996** 0.988** 0.935** 0.973**	**Catechol**	pHydroxyBenzoic acid Catechin Caffeic acid Syringic acid Benzoic acid Ferulic acid Rutin Quercetin Rosemarinic acid Neringein Cinnamic acid	0.860** −0.633* 0.931** −0.805** 0.822** −0.783** 0.989** −0.578* 0.993** 0.974** 0.962**
**Gallic acid**	pHydroxyBenzoic acid Chlorogenic acid Vanillic acid pCoumaric acid Ellagic acid oCoumaric acid Resvertol Quercetin Kampherol -------------	−0.576* −0.844** 0.896** 0.845** 0.707* 0.666* −0.872** 0.718** −0.720** -------	**pHydroxyBenzoic acid**	Catechin Caffeic acid Syringic acid Ferulic acid Rutin Quercetin Rosemarinic acid Neringein Kampherol Cinnamic acid	−0.669* 0.758** −0.947** −0.931** 0.788** −0.888** 0.839** 0.944** 0.674* 0.980**
**Chlorogenic acid**	Vanillic acid pCoumaric acid Benzoic acid oCoumaric acid Resvertol Kampherol	−0.918** −0.896** −0.834** −0.882** 0.998** 0.691*	**Benzoic acid**	Rutin oCoumaric acid Resvertol Rosemarinic acid Neringein Cinnamic acid	0.883** 0.664* −0.800** 0.834** 0.682* 0.870**
**Vanillic acid**	pCoumaric acid oCoumaric acid Resvertol Quercetin Kampherol	0.991** 0.921** −0.936** 0.801** −0.911**	**pCoumaric acid**	oCoumaric acid Resvertol Quercetin Kampherol ----------	0.942** −0.913** 0.809** −0.929** -------
**Caffeic acid**	Syringic acid Ferulic acid Rutin Quercetin Rosemarinic acid Neringein Cinnamic acid	−0.905** −0.886** 0.881** −0.801** 0.914** 0.976** 0.846**	**Syringic acid**	Ferulic acid Rutin Quercetin Rosemarinic acid Neringein Kampherol Cinnamic acid	0.953** −0.713** 0.918** −0.809** −0.901** −0.756** −0.760**
**Ferulic acid**	Rutin Quercetin Rosemarinic acid Neringein Kampherol Cinnamic acid	−0.711** 0.842** −0.759** −0.858** −0.650* −0.682*	**Quercetin**	Rosemarinic acid Neringein Kampherol	−0.583* −0.739** −0.937**
			**Rosemarinic acid**	Neringein Cinnamic acid -----------	0.968** 0.986** -------
**Rutin**	Rosemarinic acid Neringein Cinnamic acid	0.977** 0.932** 0.951**	**oCoumaric acid**	Resvertol Quercetin Kampherol	−0.882** 0.660* −0.874**
**Resvertol**	Kampherol	0.718**	**Neringein**	Cinnamic acid	0.923**

**Notes:**

* Correlation is significant at the 0.05 level (2-tailed).

** Correlation is significant at the 0.01 level (2-tailed).

**Figure 4:**
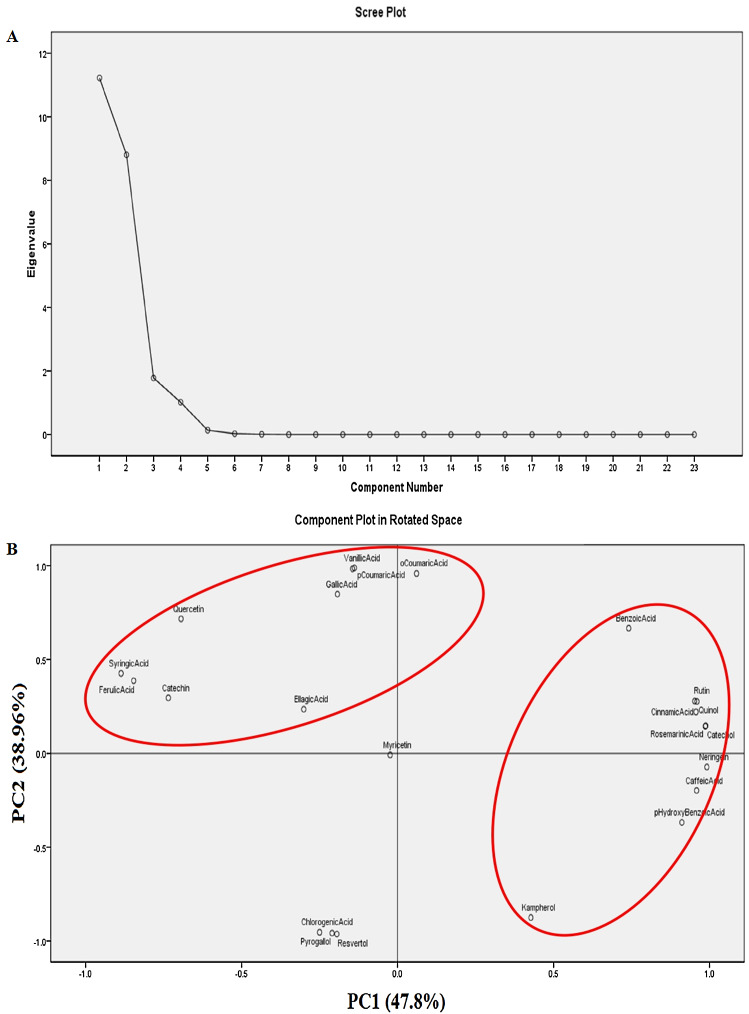
Principal components analysis (PCA) plots of the HPLC data sets (based on 23 standards) of thermally stressed seedlings of *S. lycopersicum*. (A) Scree plot showing the four extracted components with Eigen values more than one and (B) the component plot in rotated space showing the grouping of the compounds’ data set in respect to thermal stress.

Figure 4 illustrates the calculated PCA model of 23 phenolic and flavonoid compounds in response to thermal stress. Data are Gaussian or normally distributed. Four components (PC1, PC2, PC3 and PC4) were extracted by PCA. These four PCs explained 99.23% of the total variation. The first two components (PC1 and PC2) demonstrate 86.75% of total variability, displaying the difference between *S. lycopersicum* seedlings in response to thermal stress. PC1 explained ≈47.8% of the variation according to thermal stress. Meanwhile, PC2 explained ≈38.96% of the variation according to thermal stress (Fig. 4). The component matrix clarified that PC1 exhibited strong positive correlation with 8 compounds (*r > 0.83–0.99*); and moderate positive correlation with 2 compounds (*r > 0.5–0.67*). Meanwhile, PC1 demonstrated strong negative correlation with 4 compounds (*r > −0.77–0.97*). PC2 exhibited strong positive correlation with 5 compounds (*r > 0.79–0.9*); and moderate positive correlation with 3 compounds (*r > 0.52–0.55*). Meanwhile, PC2 showed strong negative correlation with 3 compounds (*r > −0.99*); and moderate negative correlation with one compound (*r > −0.68*). PC3 demonstrated moderate positive correlation with Catechin score (*r > 0.64*); and strong negative correlation with Ellagic acid score (*r > −0.89*). PC4 demonstrated moderate positive correlation with Myricetin score (*r > 0.99*).

### Total phenolic content

**Figure 5:**
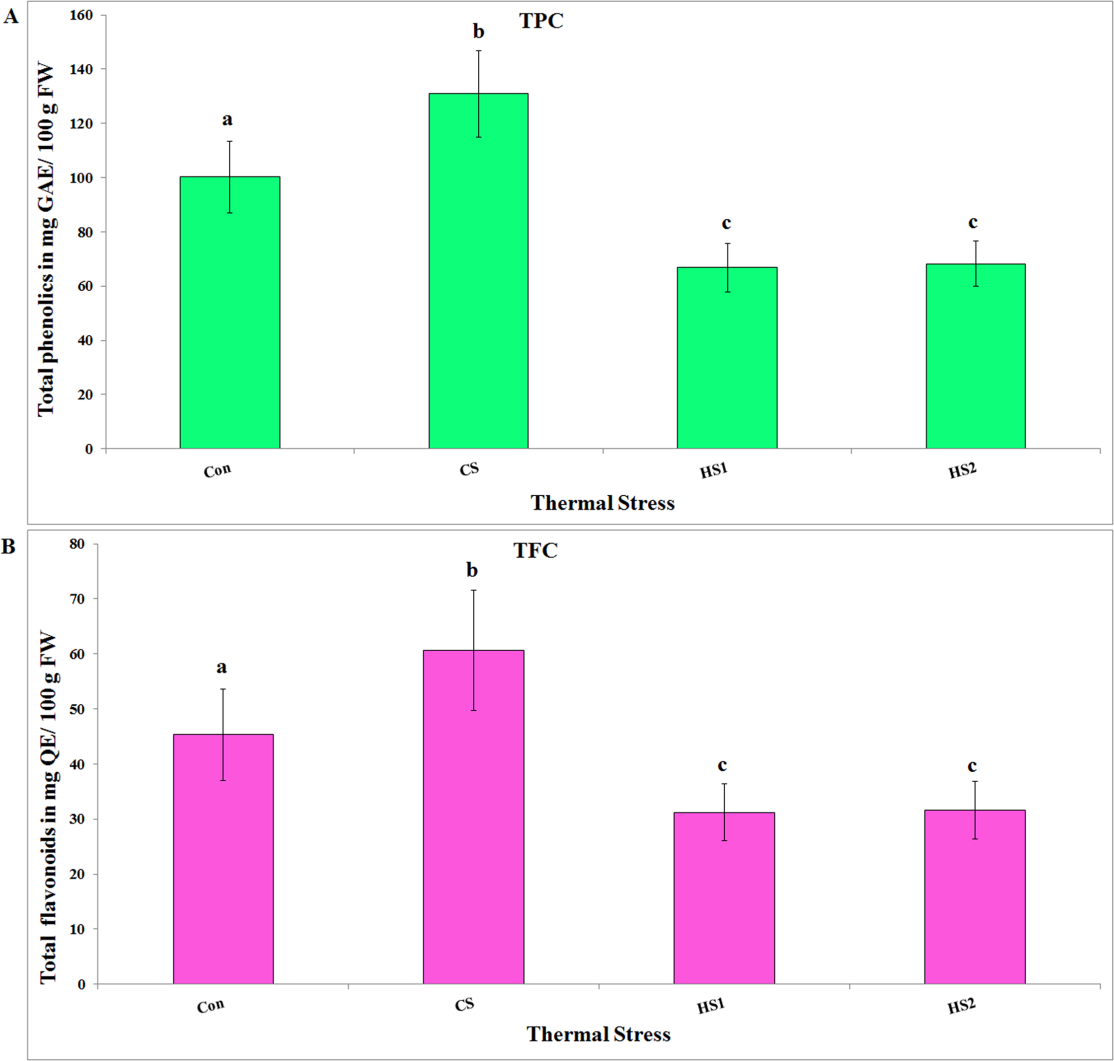
Total phenolic and flavonoid contents of the 4 ethanolic extracts of thermally stressed seedlings of *S. lycopersicum*. (A) The total phenolic contents (TPC) calculated in Gallic acid equivalent (mg GAE/100 g FW) and (B) the total flavonoid contents (TFC) calculated in Quercetin equivalent (mg QE/100 g FW). **Con, **Control; **CS,** Cold-stressed; **HS1,** First level of heat stress and **HS2,** Second level of heat stress. The lower case letters on the columns refer to significance level. Different letters refer to significant difference and the same letters refer to insignificant difference at *P < 0.05* using *Scheffè* post hoc tests.

Total phenolic contents of the 4 ethanolic extracts of thermally stressed seedlings of *S. lycopersicum* were calculated from the calibration curve of gallic acid. It is discernable that the total phenolic content varied in response to thermal stress (Fig. 5A). One-way ANOVA clarified overall significant variation with a strong positive correlation as a result of thermal stress (*df* = 3, *F* = 6.38, *P* = 0.016, *R*^*2*^ = 0. 883). Post hoc tests revealed significant difference (*P < 0.05*) when comparing Con with all treatments (CS, HS1 and HS2), CS with HS1, and CS with HS2. Conclusively, CS plants showed the highest value of TPC (≈131 mg GAE/100 g FW). Meanwhile, the lowest value (≈67 mg GAE/100 g FW) was displayed by HS1 plants (Fig. 5A).

### Total flavonoid content

To calculate the TFC of the 4 ethanolic extracts of thermally stressed seedlings of *S. lycopersicum*, quercetin calibration curve was used. It is clear that the TFC changed in response to thermal stress (Fig. 5B). Statistical analysis clarified overall insignificant variation with a strong positive correlation with the thermal stress (*df* = 3, *F* = 3.23, *P* = 0.08, *R*^*2*^ = 0.879). Post hoc analyses revealed significant difference (*P < 0.05*) when comparing Con with all treatments (CS, HS1 and HS2), CS with HS1, and CS with HS2. Discernibly, CS plants showed the highest value of TFC (≈61 mg QE/100 g FW). Whilst, HS1 plants displayed the lowest value (≈31 mg QE/100 g FW) of TFC (Fig. 5B)

### Antioxidant activity

**Figure 6:**
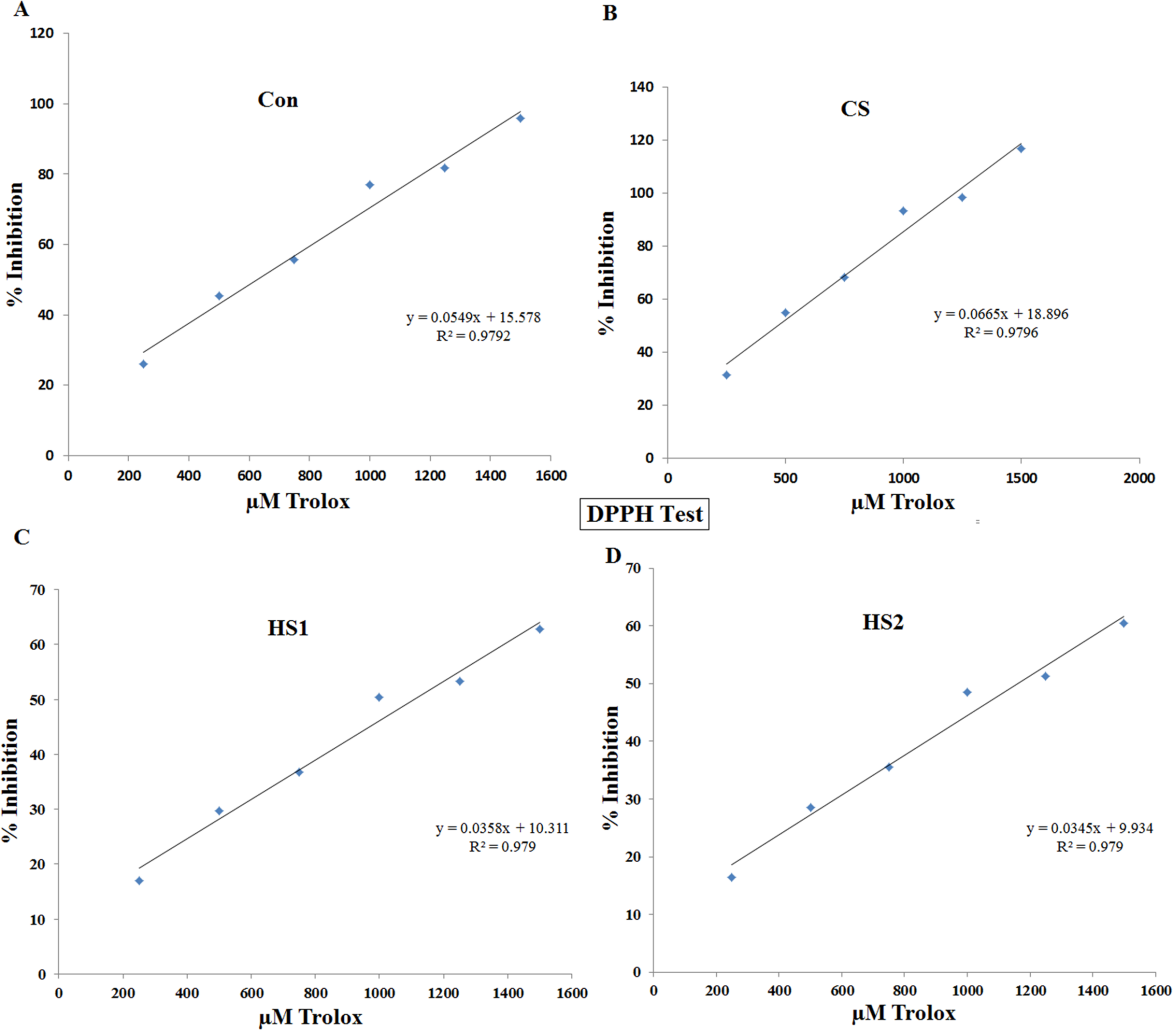
Correlation between antioxidant capacity (% inhibition) and concentration of the 4 ethanolic extracts of thermally stressed seedlings of *S. lycopersicum* by DPPH method. (A) Control (Con), (B) Cold-stressed (CS), (C) First level of heat stress (HS1) and (D) Second level of heat stress (HS2). The antioxidant capacity was calculated in terms of µM Trolox equivalent.

**Figure 7:**
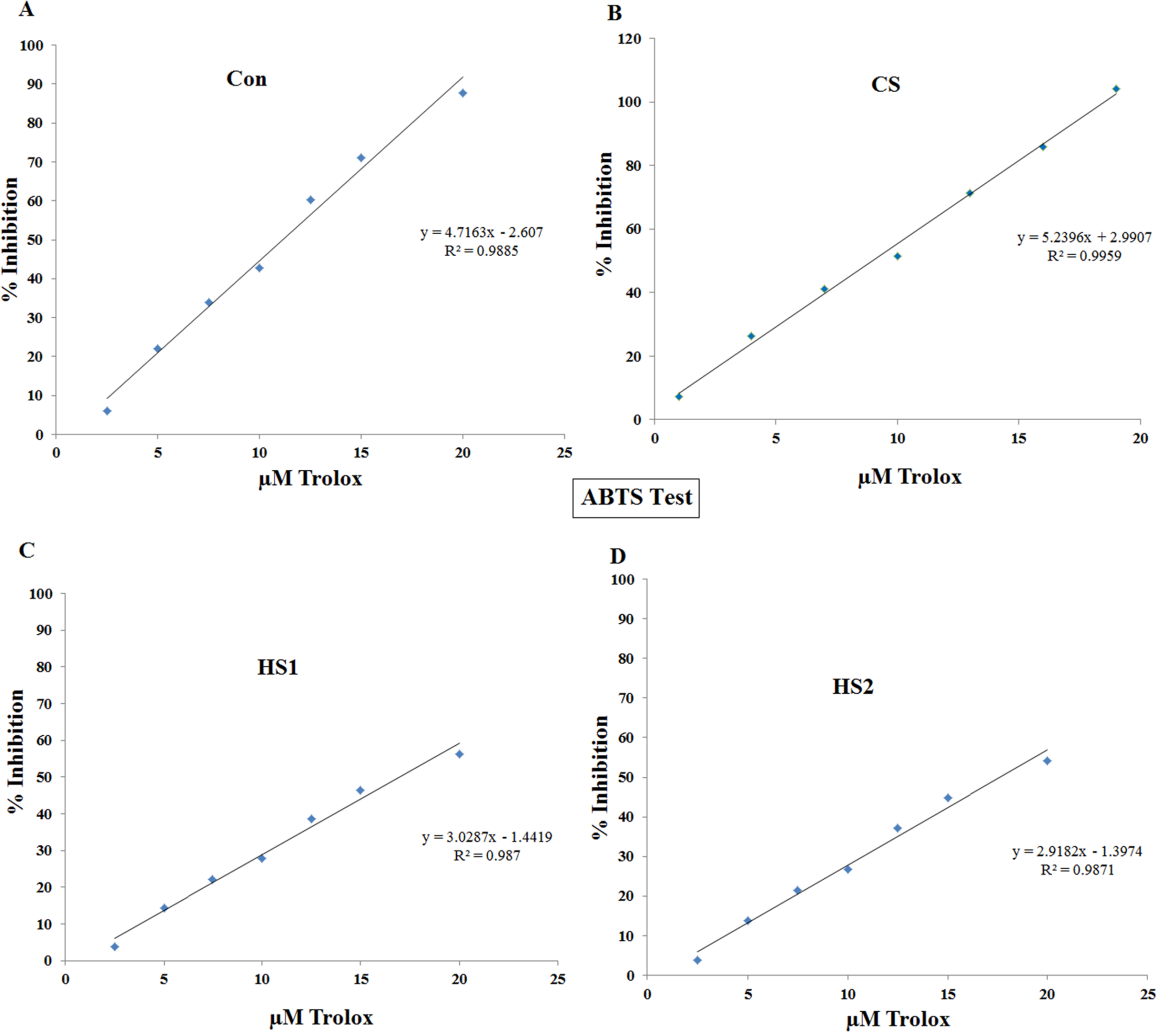
Correlation between antioxidant capacity (% inhibition) and concentration of the 4 ethanolic extracts of thermally stressed seedlings of *S. lycopersicum* by ABTS method. (A) Control (Con), (B) Cold-stressed (CS), (C) First level of heat stress (HS1) and (D) Second level of heat stress (HS2). The antioxidant capacity was calculated in terms of µM Trolox equivalent.

**Figure 8:**
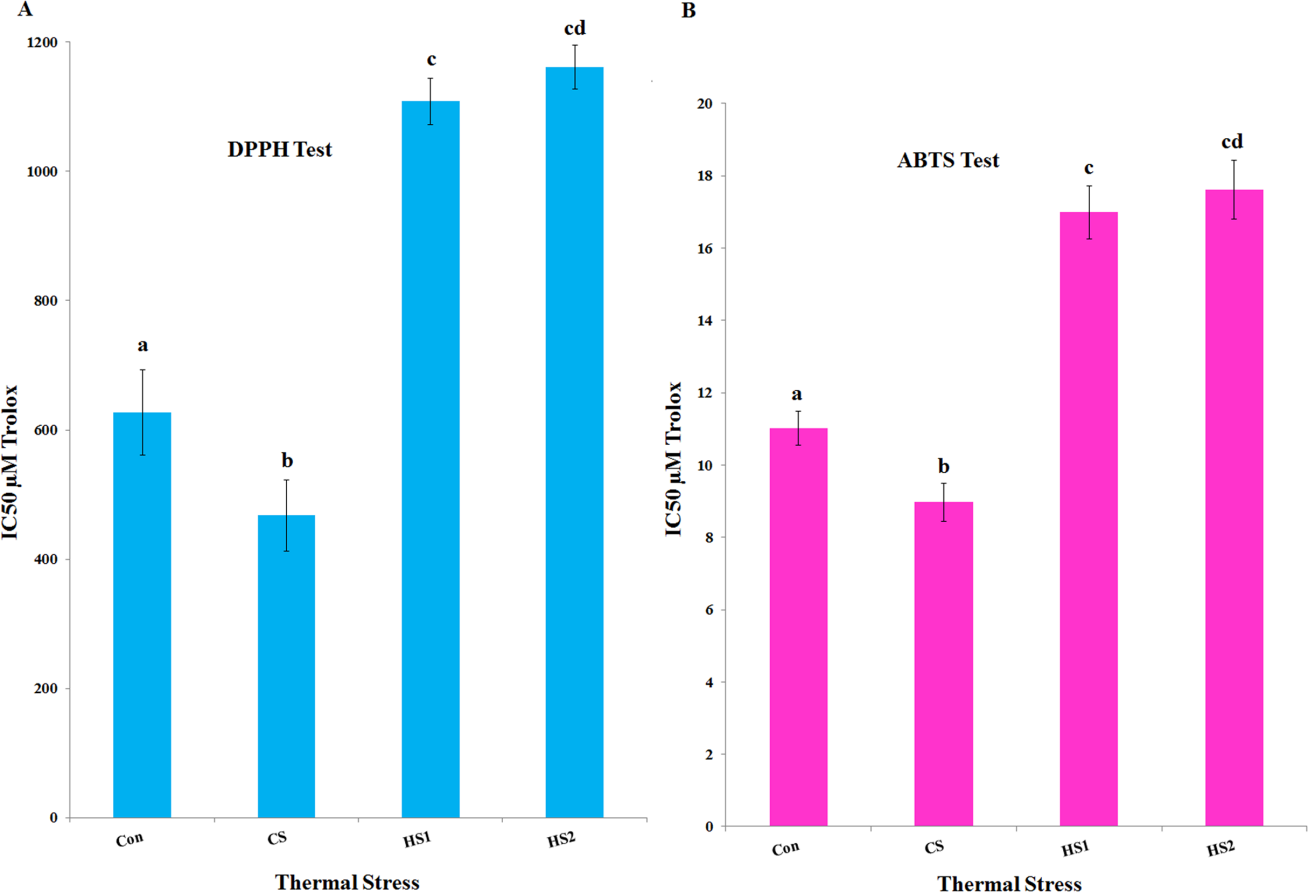
The median inhibition concentration (IC_50_s) of the 4 ethanolic extracts of thermally stressed seedlings of *S. lycopersicum* by DPPH and ABTS tests. (A) DPPH test and (B) ABTS test. **Con, **Control; **CS,** Cold-stressed; **HS1,** First level of heat stress and **HS2,** Second level of heat stress. Values of IC_50_s are calculated in µM Trolox equivalent. The lower case letters on the columns refer to significance level. Different letters refer to significant difference and the same letters refer to insignificant difference at *P < 0.05* using *Scheffè* post hoc tests.

Antioxidant activity of the 4 ethanolic extracts of thermally stressed seedlings of *S. lycopersicum*, were investigated by DPPH and ABTS methods (Figs. 6 and 7). DPPH scavenging activity was inhibited by the 4 ethanolic extracts. The percentage inhibition displayed very strong positive correlation with the concentration in cases of Con (*R*^*2*^ *= 0.989*), CS (*R*^*2*^ *= 0.999*), HS1 (*R*^*2*^ *= 0.987*) and HS2 (*R*^*2*^ *= 0.987*), as shown by Fig. 6. At the same time, ABTS scavenging activity was inhibited by the 4 ethanolic extracts. The percentage inhibition showed very strong positive correlation with the concentration in cases of Con (*R*^*2*^ *= 0.98*), CS (*R*^*2*^ *= 0.99*), HS1 (*R*^*2*^ *= 0.97*) and HS2 (*R*^*2*^ *= 0.99*), as illustrated by Figure 7. The median inhibition concentrations (IC_50_s) are calculated for both DPPH and ABTS assays. Statistical analyses revealed that the value of IC_50_ assured strong positive correlation with thermal stress in both DPPH and ABTS tests (Fig. 8). Based on 95% confidence intervals, significant difference between all treatments (*P* < 0.05) except the difference between IC_50_s of HS1 and HS2 in both DPPH and ABTS tests. The lowest values of IC_50_s were recorded in case of CS plants in both DPPH (467.73 µM TE/100 g FW) and ABTS (8.97 µM TE/100 g FW) assays. On the other hand, the highest values of IC_50_s were recorded in the case of heat-stressed plants (HS1 and HS2) in both DPPH (1108.6 and 1161.3 µM TE/100 g FW, respectively) and ABTS (16.9 and 17.6 µM TE/100 g FW, respectively) assays (Fig. 8).

## Discussion

Concerning the phytochemical complexity of plants, it is preferred to explore plant metabolome by using as much methods as we can. In such cases, justification of SMs of a plant extract is more reliable (Szabók et al., 2017; Aditya, Sil & Bhattacharjee, 2018; Isah, 2019). In addition, in vitro and in vivo temperature changes of a plant growing conditions influence the whole plant metabolism and in turn impact SMs production (Morison & Lawlor, 1999).

This study clarified that both number of identified phenolics, flavonoids and relative abundance of these compounds varied in seedlings of *S. lycopersicum* with regard to thermal stress. This increase in the intricacy of phytochemicals in seedlings of *S. lycopersicum* could be explained as impact of thermal stress. Agreeable results were presented by Scarano et al. (2020) who reported higher levels of kampherol derivatives in 8 out of 10 tomato landraces, higher levels of rutin in 3 landraces and lower in one out of 10 tomato landraces, low levels of other phenolic compounds in most landraces, and insignificant difference of chlorogenic acid among all landraces. They explained the higher polyphenol levels in some tomato landraces as a response to thermal stress (Scarano et al., 2020). In parallel, Rivero et al. (2001) indicated that thermal stress led to higher concentration of phenolic compounds in tomato plants. The accumulation of phenolic compounds in tomatoes was concluded as an acclimated mechanism against thermal stress (Rivero et al., 2001). Erge & Karadeniz (2011) determined that the amount of bioactive compounds in tomato increased, significantly, from year to year according to the climatic condition. In the current research, the higher number of signals and higher levels of phenolics and flavonoids in the case of CS plants demonstrated that the cold stress impacted seedlings of *S. lycopersicum* by increasing both the number and quantities of these compounds.

Considerable amounts (mg/kg) of the phenolic compound “Benzoic acid” were determined in Con (22.57), CS (8010.38), HS1 (3927.87) and HS2 (3995.99). Similarly, the flavonoid “Quercetin” was identified in Con (123.59), CS (2319.48), HS1 (38.83) and HS2 (344.38). The major phenolic peaks differ with each case of thermal stress (Chlorogenic acid and Resvertol in case of Con plants, Quinol, Vanillic and Benzoic acids in case of CS plants, Vanillic and Benzoic acids in case of both HS1 and HS2 plants). The major flavonoid peaks vary with each case of thermal stress (Quercetin and Myricetin in case of Con plants, Quercetin and Rutin in case of CS plants, Catechin, Quercetin and Myricetin in case of HS1 plants and Quercetin in case of HS2 plants). On the other hand, Singh et al. (2014) reported that Gallic acid (34.02 μg/g of DW) and Rutin (20.26 μg/g of DW) were the major phenolic and flavonoid compounds produced by tomato hairy root extract. Other studies reported Chlorogenic acid and Rutin as the major phenolic compounds in tomatoes (Scarano et al., 2020). Gallic acid (36.0 to 47.6 mg/100 g) and Catechin (49.3 to 61.7 mg/100 g) were reported in tomato powder, too (Kim & Chin, 2017). Integrating the previous results with ours, we concluded that the major compounds released in response to thermal stress were found to change. This change could be due to the difference in thermal stress (cold and warm), difference in plant part to be investigated and/ or due to the method of extraction as previously reported by Kim & Chin (2017).

Pearson’s correlation revealed significance in 111 pairs of compounds. Fifty three and six pairs exhibited positive strong and moderate correlations, respectively. This means that in a compound pair if one compound increases, the second increases and when it decreases the second compound decreases. Forty three and nine pairs showed negative strong and moderate correlations, respectively. This means that in a compound pair if one compound increases, the second decreases and when it decreases the other compound increases. In addition, PCA model clarified that PC1 and PC2 explained 86.75% of the total variations. These results could be interpreted referring to the component matrix as follows: the positive correlation of PC1 and 10 scores means that PC1 increases with the increase of the 10 scores (Cinnamic acid, Quinol, Rutin, Neringein, Catechol, Rosemarinic acid, pHydroxy benzoic acid, Caffeic acid, Benzoic acid and Kampherol); meanwhile in the case negative correlation, PC1 increases with the decrease of the 4 scores (Syringic acid, Ferulic acid, Quercetin and Catechin); the positive correlation of PC2 and 8 scores means that PC2 increases with the increase of the 8 scores (oCoumaric acid, Vanillic acid, pCoumaric acid, Benzoic acid, Gallic acid, Cinnamic acid, Quinol and Rutin); meanwhile in the case negative correlation, PC2 increases with the decrease of the 4 scores (Chlorogenic acid, Pyrogallol, Resvertol and Kampherol). The 59 positively correlated pairs in addition the PCA results may provide an evidence for a synergetic and cooperative way through which the antioxidants cope with the thermal stress in seedlings of *S. lycopersicum*. Worthily mentioned that a synergistic effect among antioxidants of tomatoes has been, previously, reported (Del Giudice et al., 2016).

A general significant decrease in TPC (130.9 for CS to 66.9 for HS2 mg GAE/g) and TFC (60.7 for CS to 31.2 for HS2 mg QE/g) have been observed with increasing temperature. Other literatures, reported TPC values for tomato to be 404 mg GAE/100 g DW (Toor & Savage, 2005), 34–38 mg GAE/100 g (Chang et al., 2006), 355–439 mg GAE/100 g DW (Sahlin, Savage & Lister, 2004) and 1.57–2.02 g/100 g DW of tomato powder (Kim & Chin, 2017). The varying values of TPC in different studies may be due to different stress conditions (Jochum, Mudge & Thomas, 2007; Zhao et al., 2016), tissue preparation condition (DW or FW) and/or different tissue to be studied. Alternatively, it could be a response to the growing season (Thomsen et al., 2012), or it could be due to synergism between temperature and light (Joshi, 2015). It is perceivable that the higher quantities of TPC and TFC in the case of CS plants assured that the cold stress influenced seedlings of *S. lycopersicum* by increasing quantities of both TPC and TFC. Agreeable results have reported that cold stress promoted phenolics’ production (Grifth & Yaish, 2004) and the production of high levels of chlorogenic acid was a kind of adaptation to the cold climate by the apple trees (Pérez-Ilzarbe et al., 1997). In accordance to our results, *Melastoma malabathricum* cell cultures produced higher SMs when incubated at low temperatures than at high temperature (Chan et al., 2010). Contrary to our results, heat stress created oxidative stress in the somatic embryos of *Eleutherococcus senticosus* resulting in more SMs production than cold temperature (Shohael et al., 2006). In addition, production of SMs in the *Brassica napus* callus was reported to be affected by temperatures of incubation via inducing oxidative stress (Hura et al., 2015). High levels of accumulated SMs in the hairy root cultures of *Stevia rebaudiana* and *Silybum marianum* were detected with treatment with increased incubation temperature (Kumari & Chandra, 2016; Rahimi & Hasanloo, 2016).

Antioxidant capacity (in µM TE/100 g FW) of the thermally stressed tomato plants were investigated by DPPH and ABTS. Very strong positive correlation of % inhibition with the concentration of extract were detected in all treatments (Con, CS, HS1 and HS2). This finding confirms increase–increase relationship between % inhibition and extract concentration. In terms of IC_50_s, the lowest values were determined in the case of CS plants in both DPPH (467.7 µM TE/100 g FW) and ABTS (8.97 µM TE/100 g FW) assays. Whereas the highest IC_50_ values were determined in the case of HS2 plants for both DPPH (1161.3 µM TE/100 g FW) and ABTS (17.6 µM TE/100 g FW) assays. These results provide additional evidence that cold stress (CS plants) resulted in the most potent antioxidant extract (i.e., more desirable than heat stress). The increased antioxidant activity could be due to the increased TPC and TFC in the case of CS plants. In parallel, Kim & Chin (2017) correlated the increased activity with the increased amount of TPC in the extract. The mean antioxidant activity of tomato determined by DPPH assay was estimated 52 and 49.5 μM TE/100 g DW due to climatic changes (Toor & Savage, 2005; Erge & Karadeniz, 2011). Lower values (5.4–20.9 μM TEAC/100 g) have been determined for drought and heat stressed tomato cultivars (Zhou & Yu, 2006). In addition, Zhou et al. (2019) reported dramatic increase in the antioxidant activity of heat and drought stressed tomatoes. This increase was supported by the increase in activity of the antioxidant enzymes (Zhou et al., 2019). Generally, the range of antioxidant activities of tomato cultivars was agreeable to the previously reported values of 1400–2730 μM/100 g DW (Erge & Karadeniz, 2011). The difference of antioxidant behavior of plant extracts may be due to varied relative abundance of oxidant–antioxidant compounds in the extract. Also, the addition of excess volume of extract could result in dilution of reaction system which leads to difference in the antioxidant activity.

Interestingly, the same trend of concentration in the cases of sum of both phenolics and flavonoids (CS > Con > HS1-HS2), TPC (CS > Con > HS2-HS1), TFC (CS > Con > HS1-HS2), IC_50_ using DPPH assay (CS > Con > HS1-HS2), and IC_50_ using ABTS assay (CS > Con > HS1-HS2) may provide extra evidences for the synergistic mode of action of tomato seedling to acclimate with the thermal stress. It could be concluded that each plant species, cultivar, genotype or variety has its optimal growing conditions. Deviation from the optimal range could influence the whole physiology and metabolism of the plant. Thus, the SMs production could be affected by in vivo and in vitro seasonal and regional conditions under which a plant is grown. The increasing antioxidant defenses may be considered results of an acclimation mechanism to overcome heat and cold stress, and in general unfavorable biotic and abiotic factors. Through understanding changes of SMs production and scavenging system of tomato subjected to thermal stress, SM-enhancing strategy could help in developing tolerant tomato cultivars.

## Conclusion

The integration of the obtained results in this study as a whole gives us a general figure on phytochemical changes in tomato seedlings along with controls in response to thermal stress. Overall, the phytochemical profile and antioxidant activity are strongly correlated to the thermal stress in seedlings of *S. lycopersicum*. Remarkably, CS plants induced the antioxidant defense system of *S. lycopersicum* seedlings by accumulating higher numbers and amounts of phenolic and flavonoid compounds over all treatments. The highest values of TPC, TFC, antioxidant activity (% inhibition of scavenging activity) was demonstrated by CS plants. In terms of IC_50_, CS plants exhibited the lowest values for both DPPH and ABTS tests. Perceivably, cold shock (SC plants) showed more desirable changes than heat shock, in all aspects of TPC, TFC and antioxidant activity. Finally, these results are very useful in developing strategies to produce more tolerant tomato cultivars. Additional research to reveal molecular response of tomato to such thermal stress is highly recommended.

## Supplemental Information

10.7717/peerj.11193/supp-1 Supplemental Information 1 All standards at 45 °C.

10.7717/peerj.11193/supp-2 Supplemental Information 2 All standards at 50 °C.

10.7717/peerj.11193/supp-3 Supplemental Information 3 All Standards at 25 °C.

10.7717/peerj.11193/supp-4 Supplemental Information 4 All Standards at 4 °C.

10.7717/peerj.11193/supp-5 Supplemental Information 5 Raw data of the amounts of phenolic and flavonoid compound detected by HPLC.

10.7717/peerj.11193/supp-6 Supplemental Information 6 Raw data and Calculation of ABTS & DPPH tests.

10.7717/peerj.11193/supp-7 Supplemental Information 7 Raw data of the different calculations regarding the amounts of detected compounds.

10.7717/peerj.11193/supp-8 Supplemental Information 8 Output file for ANOVA, Post Hoc tests & Pearson correlation of the compounds detected by HPLC.

10.7717/peerj.11193/supp-9 Supplemental Information 9 Output file for principal component analysis (PCA) of the compounds detected by HPLC.

## Additional Information and Declarations

### Data Availability

The following information was supplied regarding data availability: Raw data are available as Supplemental Files.

## References

[ref-1] Aditya M, Sil T, Bhattacharjee S (2018). RP-HPLC and GC-MS based identification of phenolic acids, flavonoids and hydroxyl containing compounds from one of the lead accessions of *Amaranthus hypochondriacus* L. identified on the basis of biomarkers of antioxidant potential. Basic Applied Pharmacy and Pharmacology.

[ref-2] Ainsworth EA, Ort DR (2010). How do we improve crop production in a warming world?. Plant Physiology.

[ref-3] Arnold PA, Kruuk LE, Nicotra AB (2019). How to analyze plant phenotypic plasticity in response to a changing climate. New Phytology.

[ref-4] Berini JL, Brockman SA, Hegeman AD, Reich PB, Muthukrishnan R, Montgomery RA, Forester JD (2018). Combinations of abiotic factors differentially alter production of plant secondary metabolites in five woody plant species in the boreal-temperate transition zone. Frontiers in Plant Science.

[ref-5] Camejo D, Jimenez A, Alarcon JJ, Torres W, Gomez JM, Sevilla F (2006). Changes in photosynthetic parameters and antioxidant activities following heat-shock treatment in tomato plants. Functional Plant Biology.

[ref-6] Camejo D, Martí MC, Nicolás E, Alarcón JJ, Jiménez A, Sevilla F (2007). Response of superoxide dismutase isoenzymes in tomato plants (*Lycopersicon esculentum*) during thermo-acclimation of the photosynthetic apparatus. Physiologia Plantarum.

[ref-7] Camejo D, Rodríguez P, Morales A, Dell’Amico JM, Torrecillas A, Alarcón JJ (2005). High temperature effects on photosynthetic activity of two tomato cultivars with different heat susceptibility. Journal of Plant Physiology.

[ref-8] Carvalho RF, Campos ML, Pino LE, Crestana SL, Zsögön A, Lima JE, Benedito VA, Peres LE (2011). Convergence of developmental mutants into a single tomato model system: ‘Micro-Tom’ as an effective toolkit for plant development research. Plant Methods.

[ref-9] Chan LK, Koay SS, Boey PL, Bhatt A (2010). Effects of abiotic stress on biomass and anthocyanin production in cell cultures of *Melastoma malabathricum*. Biological Research.

[ref-10] Chang C-H, Lin H-Y, Chang C-Y, Liu Y-C (2006). Comparisons on the antioxidant properties of fresh, freeze-dried and hot-air-dried tomatoes. Journal of Food Engineering.

[ref-11] De Luca V, Salim V, Atsumi SM, Yu F (2012). Mining the biodiversity of plants: a revolution in the making. Science.

[ref-12] Del Giudice R, Petruk G, Raiola A, Barone A, Montia D, Rigano M (2016). Carotenoids in fresh and processed tomato (*Solanum lycopersicum*) fruits protect cells from oxidative stress injury. Journal of Science and Food Agriculture.

[ref-13] Edreva A, Velikova V, Tsonev T, Dagnon S, Gürel A, Aktaş L, Gesheva E (2008). Stress-protective role of secondary metabolites: diversity of functions and mechanisms. General and Applied Plant Physiology.

[ref-14] Emmanuel E, Levy AA (2002). Tomato mutants as tools for functional genomics. Current Opinion in Plant Biology.

[ref-15] Erge H, Karadeniz F (2011). Bioactive compounds and antioxidant activity of tomato cultivars. International Journal of Food Properties.

[ref-16] FAOSTAT (2019). Tomato production in 2018, Crops/Regions/World list/Production Quantity.

[ref-17] Freeman BC, Beattie GA (2008). An overview of plant defenses against pathogens and herbivores. Plant Health Instructor.

[ref-18] Goyal S, Lambert C, Cluzet S, Mérillon JM, Ramawat KG, Mérillon JM, Ramawat KG (2012). Secondary metabolites and plant defense. Plant Defense: Biological Control.

[ref-19] Grifth M, Yaish MW (2004). Antifreeze proteins in overwintering plants: a tale of two activities. Trends in Plant Sciences.

[ref-20] Hänsch R, Mendel RR (2009). Physiological functions of mineral micronutrients (Cu, Zn, Mn, Fe, Ni, Mo, B, Cl). Current Opinion in Plant Biology.

[ref-21] Hartmann T (2004). Plant-derived secondary metabolites as defensive chemicals in herbivorous insects: a case study in chemical ecology. Planta.

[ref-22] Hedges LJ, Lister CE (2007). Nutritional attributes of herbs. Crop & Food Research Confidential Report No. 1891.

[ref-23] Hura K, Rapacz M, Hura T, Żur I, Filek M (2015). The effect of cold on the response of Brassica napus callus tissue to the secondary metabolites of *Leptosphaeria maculans*. Acta Physiologiae Plantarum.

[ref-24] Isah T (2019). Stress and defense responses in plant secondary metabolites production. Biological Research.

[ref-25] Jochum GM, Mudge KW, Thomas RB (2007). Elevated temperatures increase leaf senescence and root secondary metabolite concentration in the understory herb *Panax quinquefolius* (Araliaceae). American Journal of Botany.

[ref-26] Joshi N (2015). Influence of light and temperature on secondary metabolite accumulation in callus cultures of *Helicteres isora* L. IOSR Journal of Environmental Science, Toxicology and Food Technology.

[ref-27] Kim H, Chin K (2017). Evaluation of antioxidative activity of various levels of ethanol extracted tomato powder and application to pork patties. Korean Journal of Food Science of Animal Resources.

[ref-28] Kim YK, Guo Q, Packer L (2002). Free radical scavenging activity of red ginseng aqueous extracts. Toxicology.

[ref-29] Kim YS, Choi YE, Sano H (2010). Plant vaccination: stimulation of defense system by caffeine production in planta. Plant Signal Behavior.

[ref-30] Kumari M, Chandra S, Kumari M, Chandra S (2016). Secondary metabolite production in transformed cultures: Stevioside glycosides production from Stevia rebaudiana hairy root cultures. Transgenesis and Secondary Metabolism: Part of the Series Reference Series in Phytochemistry.

[ref-31] Kroymann J (2011). Natural diversity and adaptation in plant secondary metabolism. Current Opinion in Plant Biology.

[ref-32] Lin JY, Tang CY (2007). Determination of total phenolic and flavonoid contents in selected fruits and vegetables, as well as their stimulatory effects on mouse splenocyte proliferation. Food Chemistry.

[ref-33] Loyola J, Verdugo I, González E, Casaretto JA, Ruiz-Lara S (2012). Plastidic isoprenoid biosynthesis in tomato: physiological and molecular analysis in genotypes resistant and sensitive to drought stress. Plant Biology.

[ref-34] LutforRahman SM, Nawata E, Domae Y, Sakuratani T, Proft MPD, Verhoyn MNJ (2000). Effects of water stress and temperature on SOD activity, growth and yield of tomato.

[ref-35] Morison JI, Lawlor DW (1999). Interactions between increasing CO2 concentration and temperature on plant growth. Plant Cell Environment.

[ref-36] Pérez-Ilzarbe J, Hernández T, Estrella I, Vendrell M (1997). Cold storage of apples (cv. Granny Smith) and changes in phenolic compounds. Zeitschrift für Lebensmitteluntersuchung und-Forschung A.

[ref-37] Poschenrieder C, Gunsé B, Corrales I, Barcel J (2008). A glance into aluminum toxicity and resistance in plants. Science of the Total Environment.

[ref-38] Rahimi S, Hasanloo T (2016). The effect of temperature and pH on biomass and bioactive compounds production in *Silybum marianum* hairy root cultures. Research Journal of Pharmacognosy.

[ref-39] Rivero R, Ruiz J, García P, López-Lefebre R, Sánchez E, Romero L (2001). Resistance to cold and heat stress: accumulation of phenolic compounds in tomato and watermelon plants. Plant Science.

[ref-40] Sahlin E, Savage GP, Lister CE (2004). Investigation of the antioxidant properties of tomatoes after processing. Journal of Food Composition and Analysis.

[ref-41] Sato S, Kamiyama M, Iwata T, Makita N, Furukawa H, Ikeda H (2006). Moderate increase of mean daily temperature adversely affects fruit set of *Lycopersicon esculentum* by disrupting specific physiological processes in male reproductive development. Annals of Botany.

[ref-42] Scarano A, Olivieri F, Gerardi C, Liso M, Chiesa M, Chieppa M, Frusciante L, Barone A, Santinoa A, Riganob MM (2020). Selection of tomato landraces with high fruit yield and nutritional quality under elevated temperatures. Journal of Science and Food Agriculture.

[ref-43] Selmar D, Kleinwächter M (2013). Stress enhances the synthesis of secondary plant products: the impact of stress-related over-reduction on the accumulation of natural products. Plant and Cell Physiology.

[ref-44] Shohael AM, Ali MB, Yu KW, Hahn EJ, Paek KY (2006). Efect of temperature on secondary metabolites production and antioxidant enzyme activities in *Eleutherococcus senticosus* somatic embryos. Plant Cell Tissue Organ Culture.

[ref-45] Singh H, Dixit S, Verma P, Singh P (2014). Evaluation of total phenolic compounds and insecticidal and antioxidant activities of tomato hairy root extract. Journal of Agriculture and Food Chemistry.

[ref-46] Sun HJ, Uchii S, Watanabe S, Ezura H (2006). A highly efficient transformation protocol for Micro-Tom, a model cultivar of tomato functional genomics. Plant and Cell Physiology.

[ref-47] Szabók RP, Rajhárt P, Ladányi M, Németh É (2017). Stress-induced changes of growth, yield and bioactive compounds in lemon balm cultivars. Plant Physiology and Biochemistry.

[ref-48] Thomsen MG, Galambosi B, Galambosi Z, Uusitalo M, Mordal R, Heinonen A (2012). Harvest time and drying temperature effect on secondary metabolites in *Rhodiola rosea*. Acta Horticulture.

[ref-49] Toor R, Savage GP (2005). Effect of semi-drying on the antioxidant components of tomatoes. Food Chemistry.

[ref-50] Wurtzel ET, Kutchan TM (2016). Plant metabolism, the diverse chemistry set of the future. Science.

[ref-51] Xiao JB (2015). Natural polyphenols and diabetes: understanding their mechanism of action. Current Medicinal Chemistry.

[ref-52] Yang L, Wen KS, Ruan X, Zhao YX, Wei F, Wang Q (2018). Response of plant secondary metabolites to environmental factors. Molecules.

[ref-53] Yu KW, Murthy HN, Hahn EJ, Paek KY (2005). Ginsenoside production by hairy root cultures of *Panax ginseng*: infuence of temperature and light quality. Biochemical Engineering Journal.

[ref-54] Zhao YH, Jia X, Wang WK, Liu T, Huang SP, Yang MY (2016). Growth under elevated air temperature alters secondary metabolites in *Robinia pseudoacacia* L. seedlings in Cd-and Pb-contaminated soils. The Science of the Total Environment.

[ref-55] Zhishen J, Mengcheng T, Jianming W (1999). The determination of flavonoid contents in mulberry and their scavenging effects on superoxide radicals. Food Chemistry.

[ref-56] Zhou K, Yu L (2006). Total phenolic contents and antioxidant properties of commonly consumed vegetables grown in Colorado. Swiss Society of Food Science and Technology.

[ref-57] Zhou R, Kong L, Yu X, C‐O Ottosen, Zhao T, Jiang F, Wu Z (2019). Oxidative damage and antioxidant mechanism in tomatoes responding to drought and heat stress. Acta Physiologiae Plantarum.

[ref-58] Zhou R, Yu X, Ottosen C-O, Rosenqvist E, Zhao L, Wang Y, Yu W, Zhao T, Wu Z (2017). Drought stress had a predominant effect over heat stress on three tomato cultivars subjected to combined stress. BMC Plant Biology.

