# Supplementary material for: Effect of extreme temperature changes on phenolic, flavonoid contents and antioxidant activity of tomato seedlings (Solanum lycopersicum L.)
Source: PeerJ. 2021 May 12;9:e11193. doi: 10.7717/peerj.11193 (PMC8123231; doi:10.7717/peerj.11193)
Supplement: Supplemental Information 1 [file peerj-09-11193-s001.pdf]

Data File D:\DATA\PHENOLS&FLAVONOIDS2020\FSQC130-2003105.D
Sample Name: FSQC130-20

===

Acq. Operator : FSQC Lab
Acq. Instrument : Instrument 1 Location : Vial 1
Injection Date : 5/13/2020 10:29:52 AM
Inj Volume : No inj
Acq. Method : C:\CHEM32\1\METHODS\PHENOLS AND FLAVONOIDS2019NEW_LC.M
Last changed : 5/13/2020 10:23:35 AM by FSQC Lab
(modified after loading)
Analysis Method : C:\CHEM32\1\METHODS\PHENOLS_CALIBRATION_11-2019_LC.M
Last changed : 5/19/2020 11:38:25 AM by FSQC Lab
(modified after loading)
Additional Info : Peak(s) manually integrated

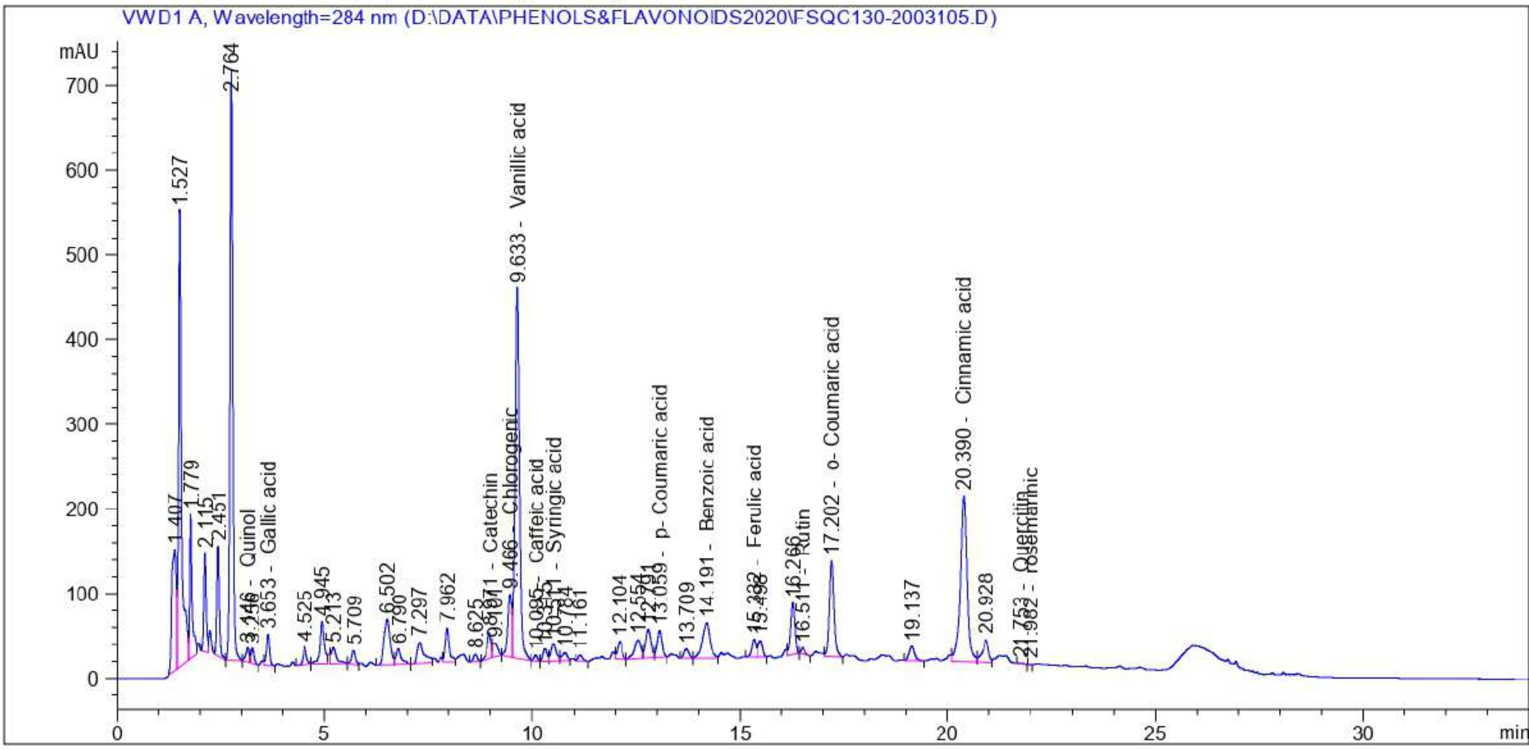

===

External Standard Report

===

Sorted By : Retention Time
Calib. Data Modified : 5/10/2020 11:34:49 AM
Multiplier: : 69.4700
Dilution: : 1.0000
Do not use Multiplier & Dilution Factor with ISTDs

Signal 1: VWD1 A, Wavelength=284 nm

RetTime [min]	Sig	Type	Area [mAU*s]	Amt/Area	Amount [mg/kg]	Grp	Name
2.900	1		-	-	-		Pyrogallol
3.146	1	BV	93.63336	1.99641e-2	129.86074		Quinol
3.653	1	VB	188.63972	6.07628e-3	79.62842		Gallic acid
