# Supplementary material for: Effect of extreme temperature changes on phenolic, flavonoid contents and antioxidant activity of tomato seedlings (Solanum lycopersicum L.)
Source: PeerJ. 2021 May 12;9:e11193. doi: 10.7717/peerj.11193 (PMC8123231; doi:10.7717/peerj.11193)
Supplement: Supplemental Information 2 [file peerj-09-11193-s002.pdf]

```
Data File D:\DATA\PHENOLS&FLAVONOIDS2020\FSQC131-2003106.D
Sample Name: FSQC131-20

    =====================================================================
    Acq. Operator   : FSQC Lab
    Acq. Instrument : Instrument 1                   Location : Vial 1
    Injection Date  : 5/13/2020 11:16:11 AM
                                                   Inj Volume : No inj
    Acq. Method     : C:\CHEM32\1\METHODS\PHENOLS AND FLAVONOIDS2019NEW_LC.M
    Last changed    : 5/13/2020 11:03:56 AM by FSQC Lab
                      (modified after loading)
    Analysis Method : C:\CHEM32\1\METHODS\PHENOLS_CALIBRATION_11-2019_LC.M
    Last changed    : 5/19/2020 11:58:07 AM by FSQC Lab
                      (modified after loading)
    Additional Info : Peak(s) manually integrated
```

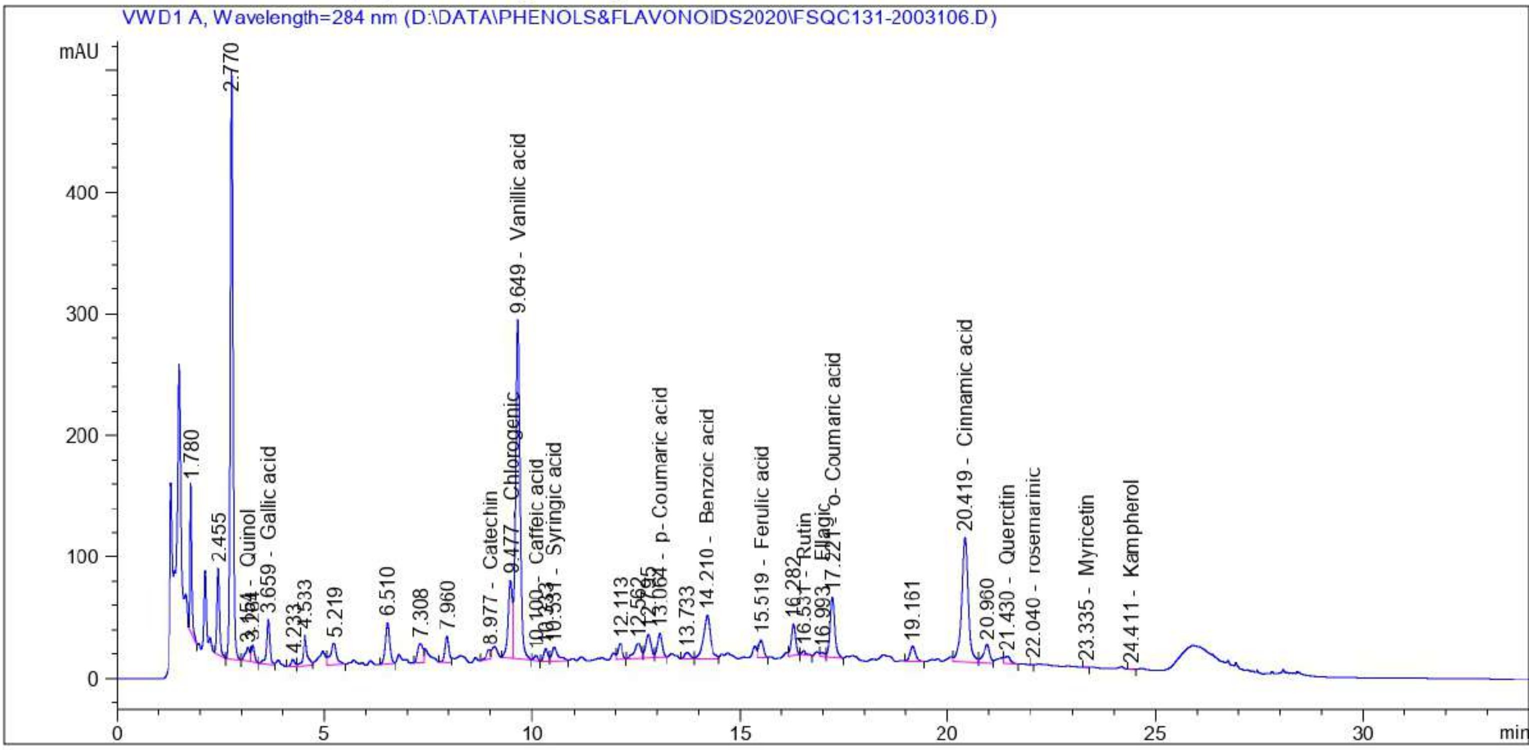


```
    =====================================================================
                         External Standard Report
    =====================================================================

    Sorted By             :      Retention Time
    Calib. Data Modified  :      5/10/2020 11:34:49 AM
    Multiplier:                    :     90.1000
    Dilution:                      :      1.0000
    Do not use Multiplier & Dilution Factor with ISTDs

    Signal 1: VWD1 A, Wavelength=284 nm
```

RetTime [min]	Sig	Type	Area [mAU*s]	Amt/Area	Amount [mg/kg]	Grp	Name
2.900	1		-	-	-		Pyrogallol
3.154	1	BV	77.13352	1.99630e-2	138.73725		Quinol
3.659	1	BV	204.73524	6.06754e-3	111.92572		Gallic acid
