# Supplementary material for: Effect of extreme temperature changes on phenolic, flavonoid contents and antioxidant activity of tomato seedlings (Solanum lycopersicum L.)
Source: PeerJ. 2021 May 12;9:e11193. doi: 10.7717/peerj.11193 (PMC8123231; doi:10.7717/peerj.11193)
Supplement: Supplemental Information 4 [file peerj-09-11193-s004.pdf]

Data File D:\DATA\PHENOLS&FLAVONOIDS2020\FSQC128-2003113.D
Sample Name: FSQC128-20

===

Acq. Operator : FSQC Lab
Acq. Instrument : Instrument 1 Location : Vial 1
Injection Date : 5/31/2020 12:33:15 PM
Inj Volume : No inj
Acq. Method : C:\CHEM32\1\METHODS\PHENOLS AND FLAVONOIDS2019NEW_LC.M
Last changed : 5/31/2020 11:47:16 AM by FSQC Lab
(modified after loading)
Analysis Method : C:\CHEM32\1\METHODS\PHENOLS_CALIBRATION_11-2019_LC.M
Last changed : 6/3/2020 10:34:11 AM by FSQC Lab
(modified after loading)
Additional Info : Peak(s) manually integrated

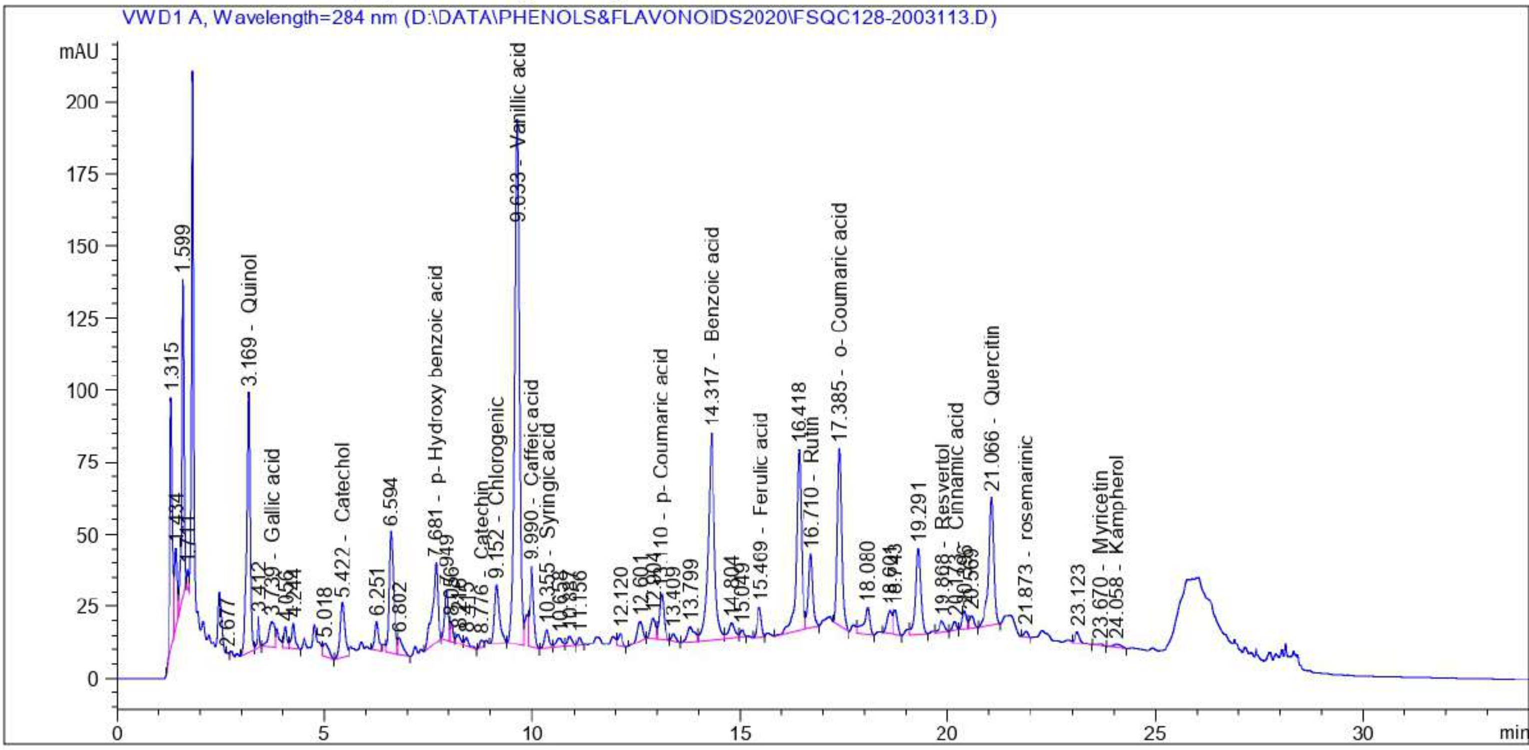

===

External Standard Report

===

Sorted By : Retention Time
Calib. Data Modified : 6/3/2020 10:34:10 AM
Multiplier: : 95.6000
Dilution: : 1.0000
Do not use Multiplier & Dilution Factor with ISTDs

Signal 1: VWD1 A, Wavelength=284 nm

RetTime [min]	Sig	Type	Area [mAU*s]	Amt/Area	Amount [mg/kg]	Grp	Name
2.900	1		-	-	-		Pyrogallol
3.169	1	BV	519.66388	1.99685e-2	992.03156		Quinol
3.739	1	BV	91.14581	6.19519e-3	53.98203		Gallic acid
